# Neuronal heterogeneity of normalization strength in a circuit model

**DOI:** 10.1126/sciadv.adv9396

**Published:** 2026-01-01

**Authors:** Deying Song, Douglas Ruff, Marlene Cohen, Chengcheng Huang

**Affiliations:** ^1^ Joint Program in Neural Computation and Machine Learning, Neuroscience Institute, and Machine Learning Department, Carnegie Mellon University, Pittsburgh, PA, USA.; ^2^ Center for the Neural Basis of Cognition, Pittsburgh, PA, USA.; ^3^ Department of Neurobiology and Neuroscience Institute, University of Chicago, Chicago, IL, USA.; ^4^ Department of Neuroscience and Department of Mathematics, University of Pittsburgh, Pittsburgh, PA, USA.

## Abstract

Neurons in higher-order visual areas integrate information through a canonical computation called normalization. The strength of normalization is highly heterogeneous across neurons, and this heterogeneity correlates with attention-mediated modulations in neural responses. However, the circuit mechanism underlying the heterogeneous normalization strength is unclear. In this work, we study normalization in a spiking neuron network model of visual cortex. Our model reveals that the heterogeneity of normalization strength is highly correlated with the inhibitory current each neuron receives. The correlation between inhibition and other synaptic inputs explains the experimentally observed dependence of spike count correlations on normalization strength. Further, we find that neurons with stronger normalization encode information more efficiently, and that networks with more heterogeneity in normalization encode visual stimuli with higher information and capacity. Together, our model provides a mechanistic explanation of heterogeneous normalization strengths in the visual cortex and sheds light on the computational benefits of neuronal heterogeneity.

## Teaser

Diverse inhibitory currents generate heterogeneous normalization that enhances visual stimulus encoding.

## INTRODUCTION

Understanding how the brain integrates and extracts information from multiple stimuli has long been a central focus in neuroscience. In visual cortex, neurons in the primary visual area respond to local features of stimuli, such as orientation and moving direction, while neurons in the higher-order visual areas have broader receptive fields and extract global features of visual stimuli. The responses of visual neurons to multiple stimuli have been well characterized by a phenomenon known as normalization, where neurons respond sublinearly. Normalization has been observed across brain regions (*1*–*3*), sensory modalities (*4*, *5*), and species (*6*–*8*) and has been regarded as a canonical computation in nervous system (*9*). Normalization has also been thought to be the fundamental mechanism through which selective attention acts to enhance neuronal responses to attended stimulus (*10*).

It has long been demonstrated that the strength of normalization is variable across neurons (*8*, *11*, *12*). Some neurons are suppressed by the addition of a nonpreferred stimulus, while some neurons show additive responses to multiple stimuli. Population recordings from multiple visual cortical areas of macaque monkeys reveal that the interactions between neurons, measured by spike count correlations, depend on their normalization strengths (*12*, *13*), which suggests that the heterogeneity of normalization is shaped by network interactions. Moreover, the heterogeneity of normalization strength is highly correlated with the neuronal heterogeneity of attention-mediated modulations in both firing rate and correlations (*8*, *11*, *13*), suggesting common mechanisms underlying normalization and attention. Therefore, an understanding of the network mechanisms underlying heterogeneous normalization strength and neuronal correlations is likely to provide insights into the neural population code of multiple stimuli and the selective modulation of stimulus information by attention.

Despite the success of the phenomenological models of divisive normalization at reproducing the firing rates of neurons under different stimulus conditions, the neurophysiological basis of normalization remains unclear. Recently, mechanistic circuit models have been developed to account for the sublinear response properties or reproduce the divisive scaling of responses (*14*–*17*). However, they focus on modeling the trial-averaged neuronal responses in homogeneous neural populations and do not consider the trial-to-trial correlations between neurons and the heterogeneity of normalization strength. Recent statistical models of normalization suggest that the strength of normalization affects the spiking variability of individual neurons and the correlations between neurons; however, they do not take into account network interactions that shape both normalization and neural correlations (*18*, *19*).

In this work, we study normalization in a two-layer network of spiking neurons, modeling the primary visual cortex (V1) and a higher-order visual area [visual area 4 (V4) or middle temporal area (MT)]. The V4/MT recurrent network produces internally generated spiking variability due to a balance of strong excitation and inhibition (*20*, *21*) (fig. S1, Aa and Ab). The neurons in our model exhibit a range of normalization strengths in response to multiple stimuli. Our model reproduces the dependence of spike count correlations on normalization strength as observed in experimental data (*12*). We identify the inhibitory current to be the major determinant of normalization strength and its relationship with spike count correlations. Further, we demonstrate that neurons with stronger normalization are more sensitive to contrast differences of visual stimuli and encode information more efficiently. Networks with more heterogeneity in normalization encode visual stimuli with higher information and capacity, demonstrating the computational benefits of neuronal heterogeneity.

## RESULTS

**Fig. 1.**
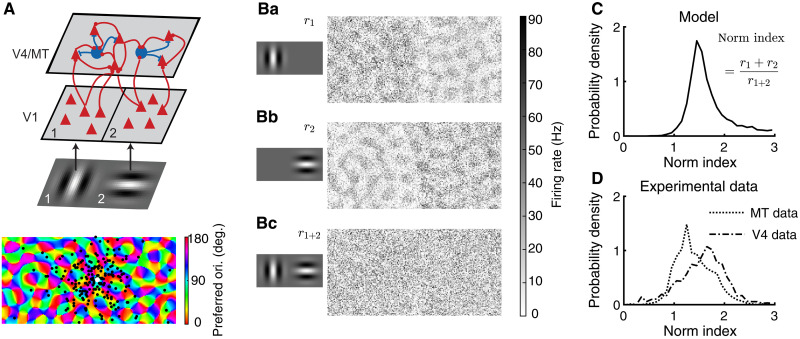
Model schematic, activation patterns, and distributions of normalization indices. (**A**) Model schematic. V4/MT area is modeled as a spatially ordered spiking neuron network of excitatory and inhibitory neurons. V1 neurons are modeled as linear-nonlinear-Poisson neurons with Gabor receptive fields. The two subpopulations of V1 neurons, V1_1_ and V1_2_, have nonoverlapping receptive fields, each centering on one Gabor image. The preferred orientations (ori.) of both V4/MT and V1 neurons are assigned according to pinwheel orientation maps (bottom). Bottom: Locations of postsynaptic excitatory neurons (black dots) of one example presynaptic excitatory neuron (white dot) in the V4/MT layer. Most of the connections are local in space, and a small portion of the connections is between similarly tuned excitatory neurons across the whole network (see Materials and Methods). deg., degrees. (**B**) Firing rate patterns of the model V4/MT neurons when one Gabor image is presented at location 1 (Ba) or location 2 (Bb) or when both images of orthogonal orientations are presented together (Bc). (**C**) The model V4/MT neurons exhibit a wide range of normalization indices. The normalization index is defined as the sum of the neuron’s firing rates in response to each image presented individually [(Ba) and (Bb)], divided by its firing rate when both images are presented together (Bc). (**D**) The normalization indices of neurons recorded from macaque V4 and MT areas [data from (*12*)].

We use a two-layer spiking neuron network model, with the feedforward layer modeling V1 neurons and the recurrent layer modeling V4 or MT area (Fig. 1A). Two Gabor images of orthogonal orientations are presented to the network. The V1 neurons are modeled as linear-nonlinear-Poisson neurons with Gabor receptive fields oriented at their preferred orientation, determined from a superimposed pinwheel orientation map (*22*). There are two populations of V1 neurons, V1_1_ and V1_2_, each of which has a nonoverlapping receptive field centering on each Gabor image, respectively.

The V4/MT layer is a recurrent network of excitatory and inhibitory neurons, each modeled as an exponential integrate-and-fire (EIF) neuron. Neurons from the V4/MT layer receive inputs from both V1 populations and respond to both images (Fig. 1B). There are two types of connections in both feedforward projections from V1 to V4/MT and recurrent projections within the V4/MT layer (Fig. 1A), following anatomical findings from visual cortex (*23*–*26*). Most of connections are local, of which the connection probability decays with distance. We choose the spatial scales of excitatory and inhibitory projections to be the same, consistent with anatomy (*23*). A small portion of connections is long range, meaning that their connection probability does not depend on distance, but they connect between similarly tuned excitatory neurons, based on the cosine similarity of their preferred orientations (see Materials and Methods). The preferred orientations of V4/MT neurons are assigned according to a superimposed pinwheel orientation map (Eq. 4), which is generated independently from the pinwheel map of the V1 neurons. The spatially dependent connections allow the V4/MT neurons to retain location information of the two images, while the tuning specific long-range connections increase the tuning selectivity and the size of receptive fields of V4/MT neurons.

The V4/MT network admits a stable and asynchronous solution with homogeneous V1 inputs, similar to the classic balanced network model (fig. S1) (*21*, *27*). The balance between strong excitation and inhibition generates Poisson-like spiking variability in individual neurons. The distribution of firing rates is lognormal due to the expansive transfer function of neurons with large input fluctuations (*28*). With orientation-tuned inputs from V1, the V4/MT model neurons capture several features of neuronal responses in visual cortex, as shown in our previous work (*29*). The tuning curves are heterogeneous with various widths and magnitudes (*30*). The spike count correlations are higher between similarly tuned neurons (*31*, *32*) and depend on stimulus orientation (fig. S2) (*33*–*35*).

### Broad distribution of normalization strengths in the model and data

We analyze the network responses to two Gabor images with orthogonal orientations, which are known to evoke the normalization mechanism in the visual cortex (*8*, *12*, *13*, *36*). Neurons’ responses to two stimuli presented together tend to be much smaller than the linear sum of the responses to each stimulus when presented individually. This phenomenon has been observed across the visual hierarchy in macaque monkeys (*12*) as well as in the primary visual cortex in cat (*36*) and tree shrew (*7*).

When presented with one image, neurons that prefer the orientation of the image are activated across the V4/MT network, resulting in local patches of active regions following the pinwheel map of orientation preference (Fig. 1, Ba and Bb). Neurons in the same spatial location of the presented image respond with higher rates because they receive more local feedforward inputs. When both images are presented together, there is no clear spatial structure of population activation pattern of firing rates (Fig. 1Bc).

We define normalization index of each neuron as the sum of the neuron’s firing rates in response to each image presented individually, divided by its firing rate when both images are presented together (Eq. 15), following the definition from our previous experimental work (*12*). A normalization index of one means that the neuron’s response to multiple stimuli is a linear summation and a normalization index larger than one means a sublinear summation. A larger normalization index means stronger normalization.

Our model V4/MT neurons exhibit a wide range of normalization indices with the majority being between one and two (Fig. 1C). The normalization indices of our model neurons span a similar range as those of neurons recorded from macaque V4 and MT areas (Fig. 1D) (*12*). The distribution of the normalization indices of the inhibitory neurons is broader than that of the excitatory neurons (fig. S3B). In addition, the normalization index of a neuron is independent from its tuning preference in both our model and data (fig. S4).

The wide distribution of normalization indices is a robust phenomenon in networks with strong recurrent connections (fig. S5). Even in random networks with no spatial or tuning dependent connections, there is a spread of normalization indices due to random connections when two input populations project to the whole network (fig. S5, A and B, magenta). The distribution widens when the two input populations project to distinct sets of target neurons (fig. S5B, cyan). In our model, the two images of orthogonal orientations activate different sets of V4/MT neurons, which is similar to the case with small overlap of input projections in the random networks (fig. S5B). In contrast, networks with weak recurrent connections produce narrow distributions of normalization strengths (fig. S6). Being able to reproduce the range of neuronal heterogeneity of normalization in our model, we next examine how neurons with different normalization strengths interact with each other.

### Spike count correlations between neurons depend on their similarity of normalization strength

The neuronal heterogeneity of normalization strength in our model is shaped by network connectivity and neuronal transfer functions. Both factors are known to determine the interactions between neurons (*20*, *37*). Next, we examine how correlations between neurons depend on neurons’ normalization indices. We use spike count correlations to measure the interactions between neurons, which is commonly used to measure the trial-to-trial cofluctuations of neuronal responses in experiments [(*38*); see Materials and Methods].

**Fig. 2.**
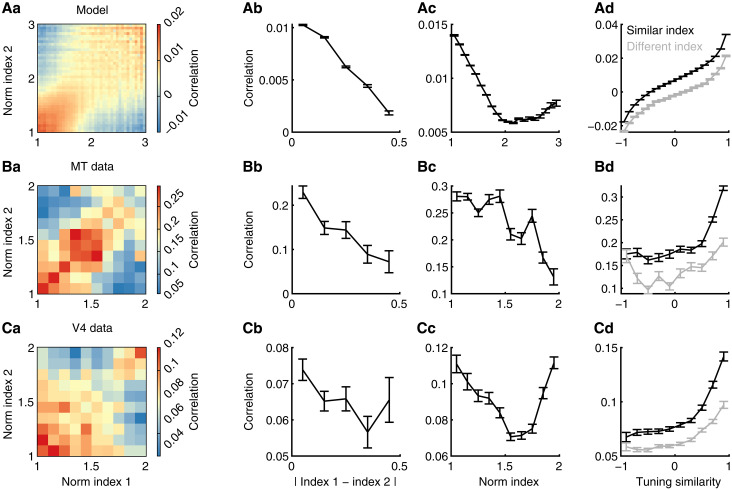
Neurons with similar normalization indices have higher spike count correlations than those with different normalization indices. (**A**) Spike count correlations between a pair of model MT/V4 neurons depend on their normalization indices. (Aa) Spike count correlations between a pair of neurons as a function of the normalization indices of the pair. (Ab) For neuron pairs of the same average normalization indices (equal to 1.5), their spike count correlations decrease with the difference in their normalization indices. (Ac) For neuron pairs of similar normalization indices (difference < 0.5), their spike count correlations decrease with their average normalization indices. (Ad) Across all levels of tuning similarity, the spike count correlations between neurons with similar normalization indices are consistently larger than those of neurons with distinct normalization indices. (**B**) Same as (A) for neuronal data recorded from the MT area (28 sessions). (**C**) Same as (A) for neuronal data recorded from the V4 area (21 sessions). Error bars represent the SEM. Data in (B) and (C) are from (*12*).

We find that spike count correlations between neurons depend on their normalization indices in the model in a way that is qualitatively consistent with the neuronal correlations measured in visual cortex (Fig. 2). First, neurons with similar normalization indices have higher spike count correlations than those with different normalization indices [diagonal versus off-diagonal elements in Fig. 2 (Aa, Ba, and Ca)]. For different neuron pairs of the same within-pair average normalization indices, their spike count correlations decrease as the difference between the normalization indices of neurons within a pair becomes larger (Fig. 2, Ab, Bb, and Cb). Second, for neuron pairs with similar normalization indices [diagonal elements in Fig. 2 (Aa, Ba, and Ca)], their spike count correlations first decrease with their average normalization indices and can increase at large normalization indices in the model and the V4 data (Fig. 2, Ac, Bc, and Cc). The same pattern of spike count correlations is also observed in neurons recorded from Macaque V1 area (fig. S7). The model also produces the same relationship between spike count correlation and normalization strengths using superimposed Gabor images in addition to two separately presented images (fig. S8). The close match of the dependence of correlations on normalization indices in our model with that in data suggests common circuit mechanisms that determine the heterogeneity of normalization. In contrast, the dependence of neuronal correlations on normalization index is very weak in networks with weak recurrent connections, emphasizing the importance of recurrent connections in determining the strength of normalization (fig. S6).

A potential explanation for the observed dependence of correlations on the similarity of normalization indices is that neurons with similar normalization indices may have more similar tuning preferences than those with different normalization indices. Several previous experimental studies have consistently demonstrated that neurons with similar tuning preferences tend to have higher spike count correlations (*31*, *32*, *34*, *35*). Therefore, tuning similarity between neurons is a potential confound that underlies the dependence of correlations on normalization indices. However, this is not the case in our model and in data. First, we find no significant correlation between the tuning preference and the normalization index of a neuron in both model and data (fig. S4). Second, we compare the spike count correlations of neuron pairs with either similar or different normalization indices across different magnitudes of tuning similarity, measured as the correlation between the tuning curves of two neurons (Eq. 17). We observe that, across all levels of tuning similarity, the spike count correlations between model neurons with similar normalization indices are consistently larger than those of model neurons with distinct normalization indices (Fig. 2Ad). We reanalyzed our experimental data and found consistent patterns in neural recordings from all three visual areas: MT (Fig. 2Bd), V4 (Fig. 2Cd), and V1 (fig. S7).

The average spike count correlation in our model is lower than that in the data. This is because the model is investigated in an asynchronous and irregular firing regime (fig. S1), which produces low spike count correlations on average, consistent with previous works (*27*, *39*). We can match the magnitude of correlations by introducing a globally correlated external input, which mimics input from other brain regions or a slow-varying neuromodulatory input (fig. S9A). The inclusion of a globally correlated input increases the average spike count correlations in our model without changing the main results (fig. S9A). An alternative mechanism for increasing the average spike count correlations in the model is by changing the network dynamical regime to a synchronous regime with wave activity, for example, by lowering the feedforward input to the inhibitory neurons (*20*, *40*). However, we find that a network in the synchronous regime produces a qualitatively different pattern of the dependence of correlations on normalization strength, with higher correlations between neurons with strong normalization than those between neurons with weak normalization (fig. S9B).

### Recurrent inhibition best explains the heterogeneity of normalization

Having demonstrated that our model successfully reproduces the distribution of normalization strength and the dependence of spike count correlations on normalization indices observed in visual cortex, we next examine the circuit mechanisms in our model that underlie the neuronal heterogeneity of normalization. We decompose the total current each V4/MT neuron receives into three components: feedforward excitation from V1 neurons, recurrent excitation from other V4/MT excitatory neurons, and recurrent inhibition from other V4/MT inhibitory neurons. We find that the population distribution of the recurrent inhibition is broader than that of the feedforward and recurrent excitation (fig. S3, Ca and Cb), which is due to the larger weight of each inhibitory connection and the more skewed distribution of the firing rates of the inhibitory neurons (fig. S3A). The large distribution of the inhibitory currents contributes to the diversity of firing rates and normalization strengths in the network.

**Fig. 3.**
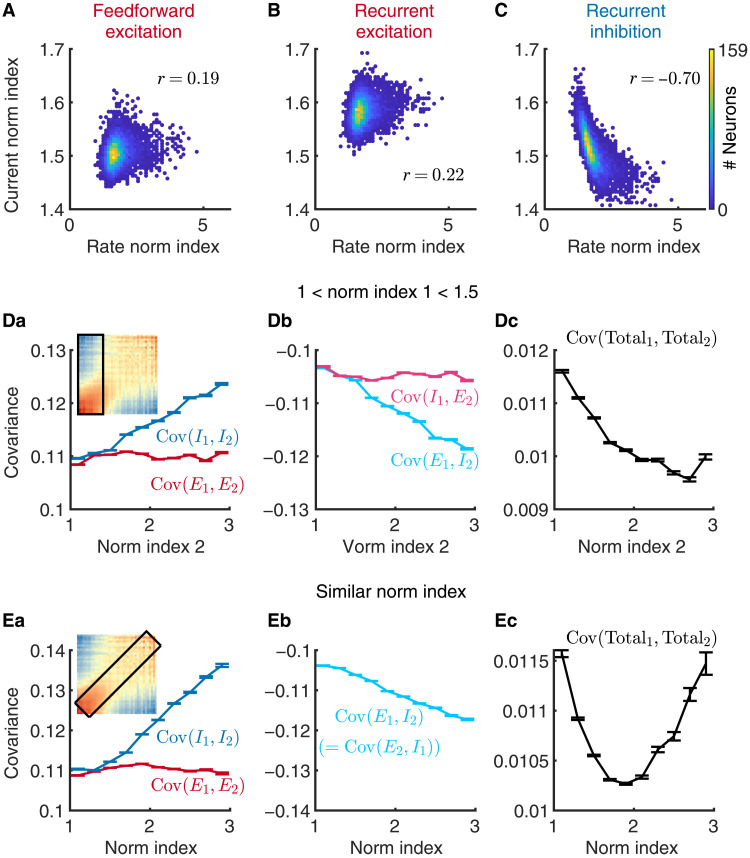
Recurrent inhibition best explains the heterogeneity of normalization. (**A**) The relationship between the normalization indices of neurons’ firing rates and those of the feedforward excitatory currents neurons receive in the model. (Pearson correlation, r=0.19, $P<10^{-4}$, N=8663) (**B** and **C**) Similar to (A), but for recurrent excitatory current [(B); r=0.22, $P<10^{-4}$] and recurrent inhibitory current [(C); r=−0.70, $P<10^{-4}$]. The normalization index of recurrent inhibitory current is strongly correlated with the firing rate normalization index. (**D**) When restricting the normalization index of neuron 1 in a pair to be between 1 and 1.5 while allowing the normalization index of the neuron 2 to vary, only the covariance components with the inhibitory currents depend on the normalization index of neuron 2 $\left[\text{Cov}\left(I_{1},I_{2}\right)\right.$ in (Da) and $\text{Cov}\left(E_{1},I_{2}\right)$ in (Db)]. Here, the excitatory current includes both feedforward and recurrent excitation. The covariance between total currents to the pair of neurons, $\text{Cov}\left({\text{Total}}_{1},{\text{Total}}_{2}\right)$, decreases with the normalization index of neuron 2 (Dc), consistent with the changes in spike count correlations [(Da), inset]. Error bars represent the SEM. Inset in (Da): Spike count correlations between a pair of neurons as a function of their normalization indices, same as in Fig. 2Aa. Black box indicates the range of normalization indices of neuron pairs analyzed in (Da) to (Dc). (**E**) Similar to (D), but for neuron pairs with similar normalization indices (difference < 0.6), indicated by the black box in (Ea) inset. Note that $\text{Cov}\left(I_{1},E_{2}\right)$ is the same as $\text{Cov}\left(E_{1},I_{2}\right)$ in (Eb), as both normalization indices of neuron 1 and neuron 2 vary together.

We find that the normalization index of each V4/MT neuron is strongly and negatively correlated with the normalization index of the inhibitory current $\left(\frac{I_{1}+I_{2}}{I_{1+2}}\right)$, defined in the same way as the normalization index of firing rate (Eq. 16; Fig. 3C). This means that neurons with stronger normalization have smaller inhibitory current normalization indices, which means higher magnitude of $I_{1+2}$ compared to that of $I_{1}+I_{2}$. Therefore, neurons with stronger normalization receive relatively more inhibition when both images are presented compared to neurons with weaker normalization (fig. S10Ac). In contrast, the correlations between the normalization indices of the firing rates and those of the feedforward and recurrent excitatory currents, respectively, are much weaker (Fig. 3, A and B). Similarly, there is a strong correlation between the firing rate normalization indices and the average inhibitory current a neuron receives when two images are presented and only weak correlations with the excitatory currents (fig. S10A). The correlation between normalization and the number of excitatory or inhibitory input connections is also weak (fig. S10B). The peak of the normalization index distribution of firing rates is aligned with that of the feedforward excitation, which is around 1.5 (Fig. 3A). Because the V1 model neurons have spontaneous rates of 5 Hz in the absence of an image in the receptive field and a population-averaged rate of 10 Hz in the presence of an image, the ratio of average feedforward drives in different stimulus conditions, $\frac{{\text{drive}}_{1}+{\text{drive}}_{2}}{{\text{drive}}_{1+2}}$, is $\frac{\left(10+5\right)+\left(5+10\right)}{10+10}=1.5$. Although the peak normalization index is mainly driven by the average feedforward input, the distribution of the rate normalization indices is much broader than that of the feedforward current. The weak correlation between normalization and feedforward excitation indicates that the normalization strength of each neuron in the model is mainly determined by recurrent connections.

The normalization strength in our network also depends on neurons’ firing rates when only stimulus 1 ($r_{1}$) or stimulus 2 ($r_{2}$) is presented. For neurons that receive less inhibition when both stimuli are presented, their normalization indices are large if they selectively respond to one of the two stimuli ($r_{1}$ large or $r_{2}$ large) and are small if both $r_{1}$ and $r_{2}$ are small (fig. S11Ac). For neurons that receive more inhibitory input when both stimuli are presented, their normalization indices are generally larger, especially when their responses to one of the two stimuli are low ($r_{1}$ or $r_{2}$ is small; fig. S11Ad). We observe similar results for the inhibitory neurons (fig. S12, Aa and Ab). The dependence of the normalization index on the firing rates $r_{1}$ and $r_{2}$ can be explained by a feedforward model with a superlinear transfer function (fig. S11; Supplementary Note 1). This analysis demonstrates the importance of stimulus overlap on the distribution of normalization indices (fig. S5). When the two stimuli activate highly overlapped neuron populations, the firing rates of each neuron for the two stimulus conditions, $r_{1}$ and $r_{2}$, are highly correlated (fig. S12, Ba and Bb). In this scenario, the network lacks neurons that respond selectively to stimulus 1 or stimulus 2, which are of strong normalization (fig. S11, Ac and Ad).

The normalization strength in our model is dependent on stimulus input. This is because the inhibition recruited to each neuron is a combination of the activation pattern of the inhibitory neurons and the connectivity. When the orientations of the pair of images are rotated, the correlation between the normalization indices calculated with the new pair of orientations and those calculated with the original pair decreases as the degree of rotation increases in our model (fig. S13A). With superimposed (plaid) images, the correlation between the normalization indices with a 45° rotation remains high (*r* = 0.61; fig. S13B), which is consistent with the experimental results [figure 3 of (*12*)].

To investigate whether the inhibitory currents also contribute to the dependence of spike count correlations on normalization indices (Fig. 2A), we compute the covariance between the excitatory and inhibitory currents that a pair of neurons receive. Let ${\text{Total}}_{i}$ be the total current neuron i receives (i=1,2) and then ${\text{Total}}_{i}=E_{i}+I_{i}$, where $E_{i}$ and $I_{i}$ are the excitatory and inhibitory current, respectively, that neuron i receives. Here, we combine both feedforward and recurrent excitation in the excitatory current, E, because the following covariance analysis yields similar patterns for both current types. The covariance between the total currents of neuron 1 and neuron 2 can then be decomposed into four components

$$\begin{array}{c} \text{Cov}\left({\text{Total}}_{1},{\text{Total}}_{2}\right)=\text{Cov}\left(E_{1},E_{2}\right)\\ +\text{Cov}\left(E_{1},I_{2}\right)+\text{Cov}\left(I_{1},E_{2}\right)+\text{Cov}\left(I_{1},I_{2}\right) \end{array}$$ (1)

Previous theoretical analysis has shown that, in balanced network models, both the excitatory and inhibitory inputs to a pair of neurons $\left[\text{Cov}\left(E_{1},E_{2}\right)\right.$ and $\text{Cov}\left(I_{1},I_{2}\right)$, respectively] are correlated, which are cancelled by the large negative correlation between the excitatory and inhibitory inputs $\left[\text{Cov}\left(E_{1},I_{2}\right)\right.$ and $\left.\text{Cov}\left(I_{1},E_{2}\right)\right]$ (*27*). In this way, the total current covariance remains small, and neurons in the balanced networks are asynchronous.

We find that only the covariance components with the inhibitory currents depend on normalization indices in our model. We focus our analysis on two cases. First, we restrict the normalization index of neuron 1 in a pair to be between 1 and 1.5 while allowing the normalization index of the neuron 2 to vary (Fig. 3Da, inset, black box region). As the normalization index of neuron 2 becomes larger and thus more distinct from neuron 1, the inhibitory current of neuron 2 becomes more correlated with the inhibitory current of neuron 1 $\left[\text{Cov}\left(I_{1},I_{2}\right)\right.$ in Fig. 3Da] and more negatively correlated with the excitatory current of neuron 1 $\left[\text{Cov}\left(E_{1},I_{2}\right)\right.$ in Fig. 3Db]. In contrast, the covariance components with the excitatory current of neuron 2 are independent of its normalization index $\left[\text{Cov}\left(E_{1},E_{2}\right)\right.$ in Fig. 3Da and $\text{Cov}\left(I_{1},E_{2}\right)$ in Fig. 3Db]. The covariance, $\text{Cov}\left(E_{1},I_{2}\right)$, becomes more negative than the increase in $\text{Cov}\left(I_{1},I_{2}\right)$, resulting in a reduction in the total current covariance, $\text{Cov}\left({\text{Total}}_{1},{\text{Total}}_{2}\right)$, as the normalization index of neuron 2 increases (Fig. 3Dc). This is consistent with the changes in spike count correlations (Fig. 2A and replicated as the inset of Fig. 3Da).

Second, we conduct the same analysis of current covariance components for neuron pairs with similar normalization indices (Fig. 3Ea, inset, black box region). We observe a similar pattern: The covariance components with the inhibitory currents, $\text{Cov}\left(I_{1},I_{2}\right)$, $\text{Cov}\left(E_{1},I_{2}\right)$, and $\text{Cov}\left(I_{1},E_{2}\right)$, increase in magnitude with normalization index, while the covariance between excitatory currents, $\text{Cov}\left(E_{1},E_{2}\right)$, is independent of normalization index (Fig. 3, Ea and Eb). Note that $\text{Cov}\left(I_{1},E_{2}\right)$ is the same as $\text{Cov}\left(E_{1},I_{2}\right)$ in this case as both normalization indices of neuron 1 and neuron 2 vary together. The total current covariance decreases initially as the normalization indices of both neurons increase and turns to increase when normalization is strong due to the large increase in the covariance between inhibitory currents, $\text{Cov}\left(I_{1},I_{2}\right)$ (Fig. 3Ec), consistent with the changes in spike count correlations (Fig. 2, Aa and Ac).

In sum, we find that neurons with stronger normalization receive more recurrent inhibition from the network. Their inhibitory currents tend be more correlated with the input currents of other neurons, which allows for better cancellation of current correlations and leads to lower spike count correlations with other neurons.

### Neurons with stronger normalization are more sensitive to contrast differences of images

**Fig. 4.**
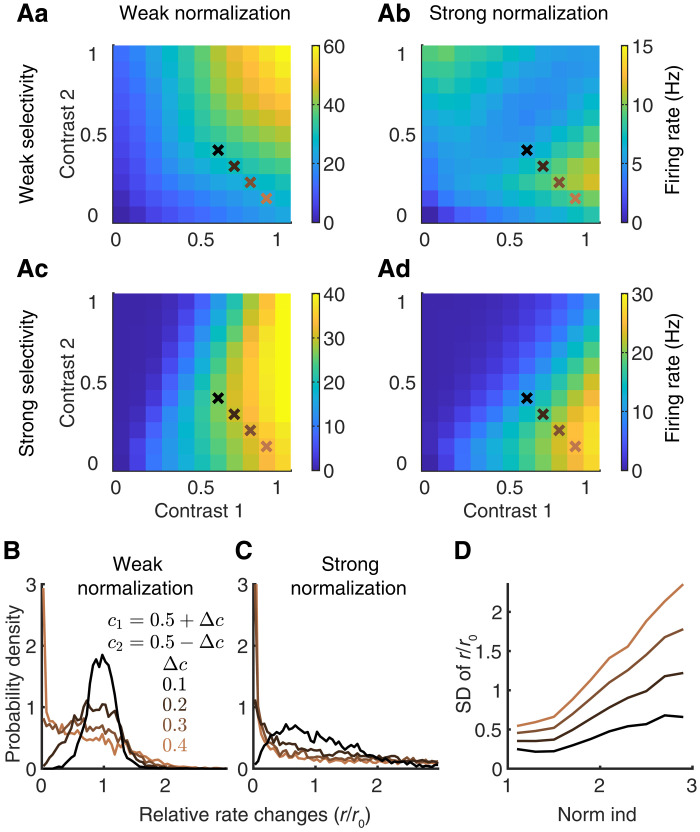
Neurons with stronger normalization are more sensitive to contrast differences of images. (**A**) Neurons exhibit diverse response tuning to different contrast combinations of two Gabor images with orthogonal orientations. (Aa) The firing rates of neurons with weak normalization and weak selectivity. (Ab) The firing rates of neurons with strong normalization and weak selectivity. (Ac) The firing rates of neurons with weak normalization and strong selectivity. (Ad) The firing rates of neurons with strong normalization and strong selectivity. Neurons that prefer stimulus 1 were selected for the results in (Aa) to (Ad). Crosses indicate the contrast combinations analyzed in (B) to (D) with the same color labels. (**B** and **C**) As contrast difference between the two images increases, the distribution of relative rate changes ($r/r_{0}$) of neurons becomes broader. The relative rate changes are neurons’ firing rates to images of a given contrast difference, Δc, divided by their firing rates to images of equal contrast (i.e., Δc=0). Neurons with strong normalization (C) have a much broader distribution of relative rate changes compared to neurons with weak normalization (B). (**D**) The SD of rate changes increases with normalization index (norm ind).

Our results, so far, have focused on the population response properties of V4/MT neurons to two images of equal contrast. We next examine the dependence of V4/MT neuron responses on the contrast of each image. We observe that neurons exhibit diverse response tuning to different contrast combinations of the two images (Fig. 4, Aa to Ad). We group neurons by their normalization indices (Eq. 15) and their response selectivity to the two images (Eq. 18). Because of symmetry, here, we only present results of neurons that prefer stimulus 1. Therefore, neurons with strong selectivity respond more strongly to stimulus 1, while neurons with weak selectivity respond similarly to both images.

The firing rates of neurons with weak normalization and weak selectivity increase linearly as the contrast of either one of the images increases (Fig. 4Aa). Thus, they respond maximally when both images have high contrast. On the contrary, neurons with strong normalization and weak selectivity are largely suppressed when both images have high contrast and respond maximally when there is only one image present (Fig. 4Ab). Neurons with strong selectivity preferentially respond when their preferred image has high contrast (contrast 1), as expected (Fig. 4, Ac and Ad). However, neurons with strong normalization are much more suppressed by increasing the contrast of their nonpreferred image (contrast 2) and respond maximally when only their preferred image is present (Fig. 4Ad). Together, neurons with heterogeneous normalization strength and selectivity preferentially respond to different contrast combinations of the two images.

We notice in the contrast dependence of responses that neurons with strong normalization are mostly active when the contrast difference between the two images is large (Fig. 4A, bottom right corners). To quantify this, we consider conditions where the average contrast of the two images is 0.5, and we gradually increase the difference between the contrasts of the two images. Specifically, we choose contrast 1 as $c_{1}=0.5+\Delta c$ and contrast 2 as $c_{2}=0.5-\Delta c$, where 2Δc is the contrast difference between the images (crosses in Fig. 4, Aa to Ad). We quantify the relative change in firing rate, $r/r_{0}$, by normalizing the firing rate of each neuron with its rate ($r_{0}$) when both images have equal contrasts, i.e., Δc=0. As Δc increases, the distribution of relative rate changes of neurons becomes broader, suggesting that some neurons become more active ($r/r_{0}>1$) while other neurons become more suppressed ($r/r_{0}<1$) (Fig. 4, B and C). Here, neurons of both stimulus preferences are included. For the same contrast difference, Δc, neurons with stronger normalization have a much broader distribution of relative rate changes compared to neurons with weaker normalization, suggesting that they are more sensitive to contrast difference (Fig. 4, B and C). The standard deviation of the distributions of relative rate changes increases with the normalization index for each Δc (Fig. 4D). This indicates that neurons with strong normalization exhibit much larger changes in their firing rates when there is a contrast difference in the two images.

### Neurons with stronger normalization encode information more efficiently

Normalization has been hypothesized to be computational advantageous because it adapts neurons’ dynamical range of responses and can increase neurons’ sensitivity to changes in stimulus. To quantify the computational benefits of normalization in our model, we measure the linear Fisher information of stimulus parameters from the activity of neurons with different normalization indices. The linear Fisher information measures the accuracy of estimating a stimulus parameter, such as contrast or orientation, from neuron population activity using an optimal linear decoder (*41*–*43*). The linear Fisher information of a stimulus parameter, s, is defined as

$$I_{F}\left(s\right)=f^{\prime }{\left(s\right)}^{T}\Sigma^{-1}\left(s\right)f^{\prime }\left(s\right)$$ (2)

where f is the tuning curve function of the neuron population with respect to s, ′ denotes differentiation with respect to s, and Σ is the covariance matrix of the population responses. Higher Fisher information means lower threshold in detecting changes in the stimulus parameter s. We focused our analysis on the information of the contrast and orientation of one image while keeping the other image at the same contrast and of an orthogonal orientation. The linear Fisher information was measured from the spike counts of the V4/MT excitatory neurons using a bias-corrected estimation [(*44*); see Materials and Methods].

**Fig. 5.**
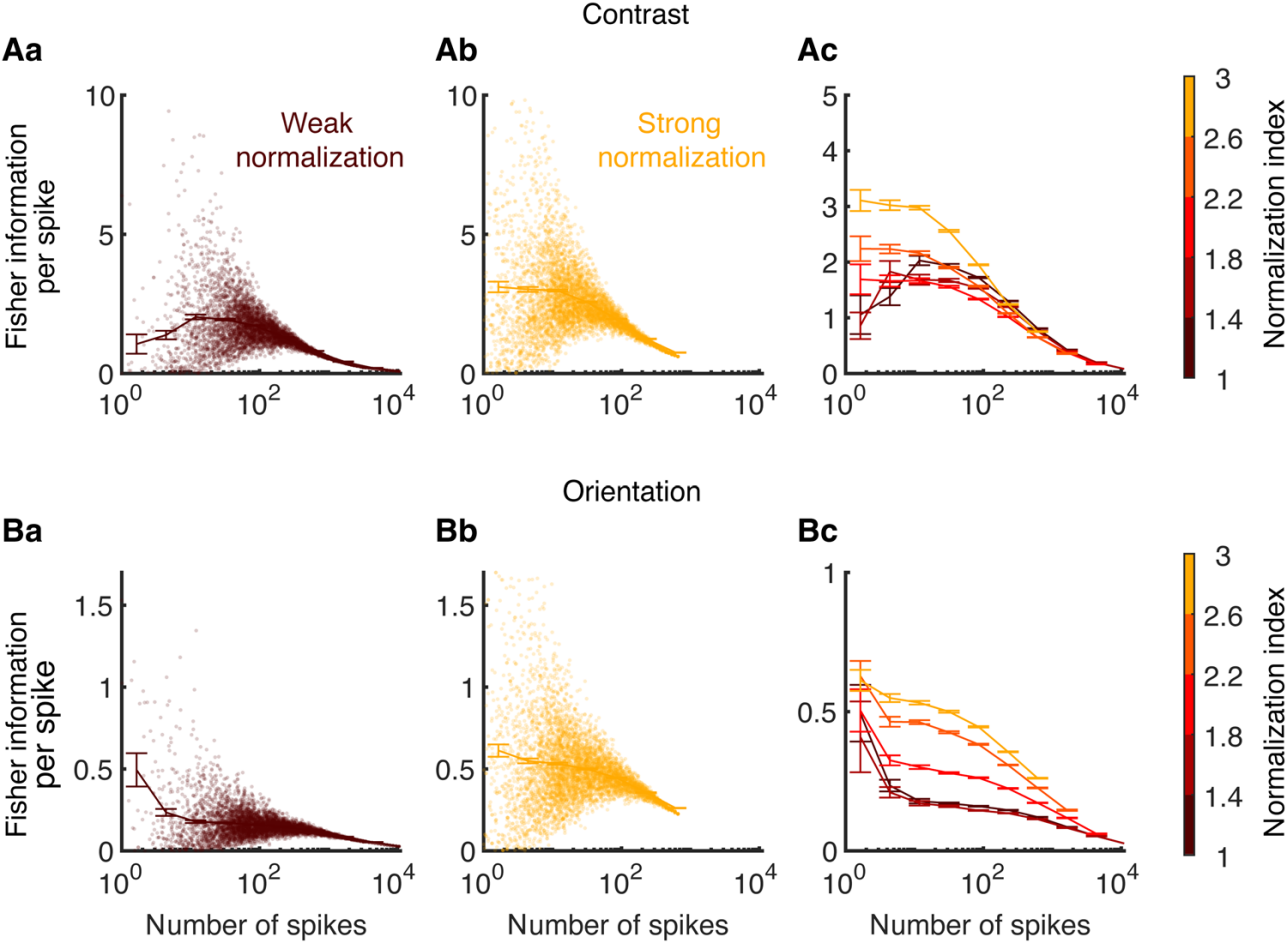
Neurons with stronger normalization encode information more efficiently. (**A**) The linear Fisher information (Eq. 2; see Materials and Methods) of the contrast of one of the two images per spike from neurons with different normalization indices. Various numbers of neurons were randomly sampled from the model V4/MT excitatory neurons whose normalization indices were within the specified range indicated by the color bar. The linear Fisher information of the sampled group of neurons was divided by the number of spikes from the sampled group of neurons during the time window used for calculating the Fisher information. (Aa) The linear Fisher information per spike as a function of the total number of spikes from the sampled neurons. Neurons with normalization indices from 1 to 1.4 were sampled. Each dot is a different sampling of a group of neurons. The solid curve is the average for each bin of the number of spikes, and the error bar is SE. (Ab) Same as (Aa) except that neurons with normalization indices from 2.6 to 3 were sampled. (Ac) The average Fisher information per spike as a function of the total number of spikes from the sampled neurons, for neurons of different ranges of normalization indices. Neurons with stronger normalization (lighter color) encode more information per spike than neurons with weak normalization (darker color). Error bars represent the SEM. (**B**) Same as (Aa) to (Ac) for the linear Fisher information of the orientation of one of the two images.

We find that neurons with stronger normalization encode more information per spike compared to neurons with weaker normalization (Fig. 5). We grouped neurons based on their normalization indices and randomly sampled a various number of neurons within each group. We then divided the Fisher information of the sampled neurons by their trial-averaged total number of spikes (see Materials and Methods). The Fisher information of contrast per spike is nonmonotonic for neurons with weak normalization with a maximum at around 12 spikes and reduces to zero as the total number of spikes increases (Fig. 5, Aa and Ac). The variance of the Fisher information per spike across samples also peaks at around 10 spikes and largely shrinks when the total number of spikes is above 100. Neurons with stronger normalization encode more information per spike when the total number of spikes is below 100 (Fig. 5Ab). This is consistent with our observation in the previous section that the firing rates of neurons with strong normalization are sensitive to contrast changes in the images (Fig. 4). The efficiency of information encoding of neurons with different normalization indices converges and decays to zero when the total number of spikes is large (Fig. 5Ac). The Fisher information of orientation shows similar trend as the information of contrast, except that the Fisher information per spike for orientation tends to decrease monotonically for neurons with weak normalization (Fig. 5B).

### Heterogeneous normalization enhances the information of image contrast and capacity for encoding natural images

**Fig. 6.**
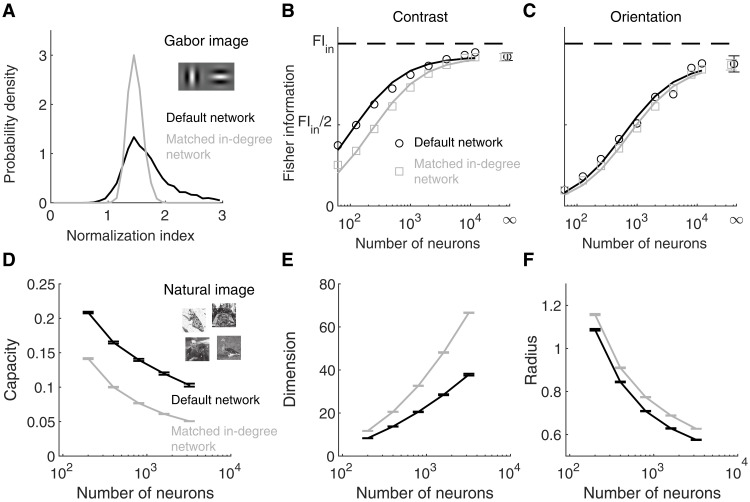
Heterogeneous normalization enhances the information of contrast in Gabor images and the manifold capacity for natural images. (**A**) The distribution of normalization indices is narrower in the network with matched in-degrees (gray) than that in the default network (black). In the network with matched in-degrees, the number of presynaptic neurons that project to each V4/MT neuron is fixed to be the same for each type of inputs (recurrent excitation, recurrent inhibition, and feedforward excitation). The default network is the same as described in Fig. 1 and analyzed in the previous figures. (**B**) The linear Fisher information of the contrast of one image as a function of the number of excitatory neurons sampled from the model V4/MT network. Open circles are the numerical estimation of the linear Fisher information (Eq. 19). The asymptotic values of the linear Fisher information at limit of large number of sampled neurons (dots at N=∞) are estimated by fitting Eq. 20, and solid curves are the fits (see Materials and Methods). Error bars are the 95% confidence intervals. Dashed line is the total amount of linear Fisher information from V1 neurons. The total input information is the same for both networks. (**C**) Same as (B) for the linear Fisher information of the orientation of one image. (**D**) The manifold capacity as a function of the number of excitatory neurons sampled from the model V4/MT neurons from the default network (black) and the network with matched in-degrees (gray). Manifold capacity measures the maximal number of linearly separable object manifolds per neuron, computed using the method from (*46*). The capacity was calculated with 50 natural images. Each point is averaged over 20 random samples of neurons. Error bars indicate SEM. (**E** and **F**) Same as (D) for the manifold dimension [(E); Eq. 22] and the manifold radius [(F); Eq. 23].

Last, we compare the information content in networks with different amount of heterogeneity in normalization. We have shown that the normalization index of a model neuron is strongly correlated with the inhibitory current it receives (Fig. 3, A to C). To reduce the heterogeneity in normalization, we constructed a control network with the same number of input connections (i.e., in-degree) to each V4/MT neuron, including both local and long-range connections, so that neurons receive roughly the same magnitude of currents. All the other parameters, such as the connection weights and the spatial spreads of connections, were kept the same as the default network. Matching the in-degrees in a homogeneous network without spatial or tuning dependent connections leads to similar firing rates in neurons from the same cell-type population (excitatory or inhibitory) (*39*). Matching the in-degrees in our spatial network also largely reduces the spread of normalization indices (Fig. 6A). The remaining heterogeneity in normalization is partly due to the tuning selectivity of neurons and the spatial arrangement of the pinwheel orientation map.

As the number of sampled V4/MT neurons increases, the Fisher information of the sampled neurons increases and approaches the upper bound of the total input information from V1 neurons (Fig. 6, B and C). The information of image contrast is higher in the default network than that in the network with matched in-degrees, which has less heterogeneity in normalization (Fig. 6B). The information difference between the two networks reduces as the number of sampled neurons increases and the information curves from both networks saturate to a similar level close to the upper bound of input information. This again demonstrates the efficiency of neural coding in networks with heterogeneity, that is, networks with more heterogeneity of normalization encode more information of contrast with a smaller number of neurons.

The information of orientation is very similar in both networks with different amounts of normalization heterogeneity across various numbers of sampled neurons, with the information from the default network being slightly higher (Fig. 6C). This suggests that heterogeneous normalization is more beneficial for encoding contrast than orientation in our network.

We further investigate the potential advantages of heterogeneity in normalization with natural images. We calculated the manifold capacity in the default network and the network with matched in-degrees, using the method from (*45*, *46*). Each manifold is the population responses to one natural image over many trials. The capacity of a network is the maximal number of linearly separable object manifolds per neuron (see Materials and Methods). We find that the manifold capacity is higher in the default network with more heterogeneity than in the network with matched in-degrees, which has less heterogeneity (Fig. 6D). The difference in capacity is present over different numbers of sampled neurons. The higher capacity in the default network is due to lower dimension and smaller radius of each manifold compared to those in the network with matched in-degrees, which make linear separation of manifolds easier (Fig. 6, E and F). These results demonstrate the benefits of heterogeneous normalization in the encoding capacity of natural images.

## DISCUSSION

Normalization mechanism has often been used to describe neurons’ sublinear responses to multiple stimuli in visual cortex (*1*, *8*, *13*, *47*, *48*). However, neurons exhibit diverse response patterns in their integration of multiple stimuli; some neurons show facilitated responses to two stimuli, while some neurons are strongly suppressed by an addition of a nonpreferred stimulus (*6*, *8*, *12*, *49*). The mechanism underlying the neuronal heterogeneity of normalization and its contribution to neural coding is not well studied. In this work, we analyzed response properties of a recurrent network of excitatory and inhibitory spiking neurons modeling the visual cortex. Our model neurons exhibit a range of normalization strengths that is consistent with experimental data (*12*). We find that a neuron’s normalization strength is strongly correlated with the relative magnitude of inhibitory current that it receives. In addition, our model reproduces the relationship between normalization strength and the spike count correlations between pairs of neurons observed in experimental data, which can be explained by the correlation between inhibitory current and other synaptic inputs. Further, we demonstrate that model neurons with different normalization strengths and selectivity respond to different combinations of the stimulus contrasts. Model neurons with stronger normalization are more sensitive to the contrast difference of images and encode more stimulus information per spike. Last, we show that neuronal heterogeneity can be beneficial for coding, as networks with more heterogeneity encode with higher information and manifold capacity.

### Strong recurrent coupling and the excitation-inhibition balance

We find that strong recurrent coupling among the excitatory and inhibitory neurons and distinct sources of feedforward inputs are important for generating a wide range of heterogeneity of normalization strength. In networks with weak recurrent coupling, the distribution of normalization indices is narrow and the spike count correlations between neurons only weakly depend on their normalization strengths (fig. S6). This suggests that strong recurrent connections amplify the heterogeneity in neuron responses to multiple inputs. Increasing the variance of connection weights or the in-degrees of inhibitory connections can also increase the heterogeneity of normalization strength when the mean recurrent coupling is weak in the network (figs. S14 and S15). However, they tend to generate more neurons with weaker normalization. The networks with broad in-degrees also do not reproduce the dependence of correlations on normalization strength (fig. S15). In networks with strong recurrent coupling and disordered connections (i.e., no spatial or tuning dependence of connections), neurons exhibit a range of normalization strengths when the network receives two sources of feedforward inputs (fig. S5). The distribution of normalization strengths broadens as the two sources of inputs target more distinct populations of neurons. The range of normalization strengths is similar to that of the detailed circuit model of visual cortex where the two images of orthogonal orientations activate largely nonoverlapped groups of neurons in the V4/MT network (Fig. 1). Our results are consistent with a recent finding where models with strong recurrent coupling explain the large distribution of rate changes in monkey visual cortex induced by optogenetic stimulation (*50*).

The balanced network model (*21*) has been successful at explaining the genesis of neuronal variability and the close relationship of excitation and inhibition observed in experiments in cortex (*51*–*53*). However, balanced networks produce a linear relationship between input and output rate, which has been considered as a limitation for complex computation (*54*). Recent theoretical work suggests that nonlinear computation can be achieved in balanced networks when some neurons are silenced by excess inhibition, creating a local imbalance in currents (*55*). They show that a network can produce sublinear summation, if individual stimulus silences a group of neurons when presented alone (*55*). Our finding is different from this work in that we analyzed neurons with positive rates in all three stimulus conditions (stimulus 1 alone, stimulus 2 alone, or both). In other words, neurons in our model do not need to be silenced in one stimulus condition to show sublinear summation. Consistent with the previous work, our results demonstrate that individual neurons can exhibit diverse and nonlinear response functions to multiple stimuli, although the population averaged rate remains mostly linear (Fig. 4, Aa to Ad).

### Network mechanisms of normalization

Although the normalization phenomena have been widely observed in visual cortex, as well as in other brain regions, the source of normalization has been under debate. It was initially hypothesized that the divisive form of normalization can be implemented by shunting inhibition (*56*). However, it has been shown that shunting inhibition alone results in a subtractive, not divisive, modulation of firing rates (*57*). Instead, divisive gain modulation can be achieved by modulating both excitatory and inhibitory inputs in a balanced manner (*57*). Later experimental findings suggest that it is the excitation, rather than inhibition, that underlies the sublinear responses of neurons because both excitation and inhibition are suppressed with an addition of nonpreferred stimulus (*58*). Recent experiments suggest that a feedforward mechanism is sufficient to account for the sublinear responses of neurons in primary visual cortex without invoking a recurrent mechanism (*6*, *59*), although mechanisms for higher-order visual areas are not studied. In our model, we find that the normalization strength of a neuron is highly anticorrelated with the normalization strength of the inhibitory current that it receives and is only weakly correlated with that of the feedforward and recurrent excitatory currents (Fig. 3, A to C). Moreover, the covariance with the inhibitory current also explains the relationship between spike count correlation and normalization. Neurons with stronger normalization receive relatively more inhibitory currents, which cancel out more correlation in their currents, making those neurons less correlated with other neurons. Our results are consistent with previous theoretical work that suggests that the population firing rate patterns in cortical circuits are primarily determined by inhibitory currents (*60*). Together, our results emphasize the role of inhibition in determining the normalization strength and neuronal correlations.

Numerous experimental works have demonstrated that the neural mechanisms of normalization and selective attention are closely related (*8*–*10*, *61*, *62*). In particular, the neuronal heterogeneity of attentional modulation in firing rates is highly correlated with the neuronal heterogeneity of normalization, meaning that neurons that demonstrate stronger normalization are also more modulated by attention (*8*, *11*). In addition, the spike count correlations among neurons with stronger normalization are also more modulated by attention (*13*). Both experimental and modeling works suggest that inhibitory neurons are more targeted by attention than excitatory neurons, which could stabilize population dynamics and reduce neural correlations (*20*, *63*–*65*). Our finding of the strong correlation between normalization strength and inhibitory current (Fig. 3C) suggests that inhibition may be the unifying mechanism that relates the neuronal heterogeneity of normalization and attentional modulation. Future extension of our model is needed to explore the interplay between normalization and attention mechanisms in enhancing the neural representation of attended stimuli.

Several network models have been proposed to explain the normalization mechanism (*14*, *17*, *66*). One of them, the stabilized supralinear network (SSN) model (*14*, *35*), is closely related to our model. The SSN model also has strong recurrent excitation that is stabilized by inhibitory feedback, and the recurrent connections depend on tuning similarity. The SSN model reproduces the sublinear summation of neuronal responses to two stimuli and the quenching of neuronal variability by stimulus contrast. The key differences between our model and the SSN model are that our model consists of spiking neurons instead of rate units and that the population rate of our model do not show strong saturation as input strength increases. Because both our model and the SSN model are in the inhibition-stabilized regime and we identify inhibition as the main determinant of the normalization heterogeneity in our model, we conjecture that the SSN model may share common mechanisms for generating heterogeneous normalization strength. To test this hypothesis, we implemented the two-dimensional version of the SSN model with probabilistic connections to introduce heterogeneity (fig. S16) (*14*). We found that the normalization index of a neuron strongly depended on the neuron’s preferred orientation in the SSN model, which was not observed in our experimental data and the spiking network model (compare fig. S16B with fig. S4). There was a similar relationship between spike count correlations and normalization indices in the SSN model; however, the relationship was absent after we matched for the distribution of tuning preferences across normalization indices (fig. S16C).

### Model limitations and alternative mechanisms of normalization heterogeneity

While our findings offer insights into the circuit mechanisms underlying heterogeneous normalization strengths in the visual cortex, several limitations need to be considered. First, our model primarily investigates spatially separated visual stimuli, whereas many experimental studies on normalization use superimposed (plaid) stimuli (*1*, *12*, *36*). Although the use of separated stimuli facilitates the study of spatial attention and its relationship with normalization mechanisms, the use of different visual stimuli may involve different circuit-level mechanisms. We demonstrate that superimposed stimuli in our model produce qualitatively the same results (fig. S8). Second, both surround suppression and cross-orientation interactions are well-documented in the visual cortex (*67*). The visual stimuli in our model are different from a typical center-surround stimulus in that the higher-order area in our model receives direct feedforward inputs from both images. In other words, both images are within the center receptive field of the V4/MT model neurons. Recent experimental and modeling work demonstrates that neurons from mouse primary visual cortex have facilitated responses when the center and surround images have orthogonal orientations, which is mediated by the disinhibitory circuit of vasoactive intestinal peptide–expressing and somatostatin-expressing interneurons (*68*). Future models incorporating different inhibitory interneuron subtypes may help generalize our findings and provide a more comprehensive account of normalization mechanisms.

Besides recurrent inhibition identified in our model, a number of other mechanisms could provide alternative explanations for the heterogeneous normalization strengths in the visual cortex. For example, colocalized and distributed synaptic inputs could undergo different nonlinear dendritic summations, supralinear or sublinear (*69*), resulting in different normalization indices. Similarly, variability in intrinsic neuronal properties could also lead to heterogeneous normalization indices. Electrophysiology recordings from slice and culture have shown that neuronal adaptation amplitude and decay constant can differ more than 10-fold across cortical cell classes and even among neurons of the same nominal type (*70*). The differential adaptation rates can either suppress or enhance neural responses to a complex stimulus, as shown in recordings from macaque inferior temporal cortex (*71*). In addition, different ion channel densities and axon initial segment morphology could lead to differences in neuronal excitability (*72*), resulting in different nonlinear response functions.

### Computational benefits of normalization and the neuronal heterogeneity in normalization

Past work has proposed several computational benefits of normalization [summarized in review (*9*)]. For example, by adapting neurons’ response range based on background input, neurons can remain sensitive to small changes in stimulus. This is most evident for retinal neurons that need to respond to light intensities over a range of several orders of magnitude (*73*). In visual cortex, it has been shown that divisive normalization can reduce the statistical redundancy present in natural images (*74*). Divisive normalization can also implement marginalization in the framework of probabilistic population code (*43*). Complementary to these works, our results reveal additional benefits of normalization, that is, neurons with stronger normalization encode more stimulus information per spike (Fig. 5).

In addition, we demonstrate that the neuronal heterogeneity of normalization contributes to coding. Past works have shown that cellular heterogeneity, such as in spiking threshold or excitability, can increase network responsiveness (*75*), improve network resilience to changes in modulatory inputs (*76*), and enhance the mutual information between stimulus and neural responses (*77*, *78*). Heterogeneity in neuronal timescales can also improve learning in tasks with rich intrinsic temporal structure (*79*). Our work is different from these works in that our model neurons are homogeneous in terms of their cellular properties, and the heterogeneity in their response properties is generated by network interactions. The heterogeneous normalization strength allows the neural population to encode different contrast combinations of the two images (Fig. 4). In networks with less heterogeneity of normalization, more neurons are needed to encode the same amount of information (Fig. 6B). Therefore, neuronal heterogeneity in normalization improves the efficiency of information coding. Heterogeneous normalization also increases the network capacity to encode natural images by reducing the dimensionality and radius of the neural response manifold for each image (Fig. 6, D to F). We find that heterogeneity has a larger impact on the information of image contrast than on the information of orientation. Future work is needed to investigate how neuronal heterogeneity affects population representational geometry of multiple stimulus features in circuit models.

## MATERIALS AND METHODS

### Spiking neuron network model of visual cortex

The model consists of two layers of spiking neurons, modeling the primary visual cortex (V1) and a higher-order visual area (V4 or MT), respectively (Fig. 1A). Neurons from the two layers are arranged on a uniform grid covering a rectangle of size Γ=[0,2]×[0,1]. There are 5000 excitatory neurons in the V1 layer, 20,000 excitatory neurons and 5000 inhibitory neurons in the V4/MT layer.

#### V1 model neurons

V1 neurons are modeled as linear-nonlinear Poisson spiking neurons, following previous models (*29*, *80*). V1 neurons are divided into two populations, V1_1_ and V1_2_, each of which has a nonoverlapping receptive field centering on each Gabor image, respectively. Neurons located at the left half of the rectangle Γ ([0,1]×[0,1]) have receptive fields centered at and with the same size as image 1, while those located at the right half of the rectangle ([1,2]×[0,1]) have receptive fields centered at and with the same size as image 2. The receptive field of a neuron from population k (k=1,2) is modeled as a Gabor filter

$$\begin{array}{c} F_{i}^{\left(k\right)}\left(x,y\right)=\text{exp}\left\{-\frac{1}{2\sigma^{2}}\left[{\left(x-x_{k}\right)}^{2}+{\left(y-y_{k}\right)}^{2}\right]\right\}\\ \text{cos}\left\{\frac{2\pi }{\lambda }\left[\left(x-x_{k}\right)\text{cos}\theta_{i}+\left(y-y_{k}\right)\text{sin}\theta_{i}\right]\right\} \end{array}$$ (3)

where the subscript i denotes the neuron’s index, σ=0.2 is the SD of the Gaussian envelope, λ=0.6 represents the wavelength of the sinusoidal factor, x and y are the coordinates of the neuron, $\left(x_{k},y_{k}\right)$ is the center of the receptive field $\left[\left(x_{1},y_{1}\right)\right.$ = (0.5, 0.5) for V1_1_ neurons and $\left(x_{2},y_{2}\right)=\left(1.5,0.5\right)$ for V1_2_ neurons], and $\theta_{i}$ is the preferred orientation of neuron i. The preferred orientation of each neuron was assigned according to a pinwheel orientation map generated with supplementary materials equation 20 from (*22*). The preferred orientation at (x,y) is $\theta_{i}\left(x,y\right)=\text{angle}\left[z\left(x,y\right)\right]/\left(2\pi \right)$ and

$$\begin{array}{cc} z\left(x,y\right)= & \sum_{j=0}^{n-1}\text{exp}\left(i\frac{2\pi }{\Lambda }\left\{l_{j}\left[\text{cos}\left(j\pi /n\right)x+\text{sin}\left(j\pi /n\right)y\right]+\phi_{j}\right\}\right),\\ & n=30 \end{array}$$ (4)

where Λ=0.125 is the average column spacing, $l_{j}=\pm 1$ is a random binary vector, and the phase $\phi_{j}$ is uniformly distributed in [0,2π].

Spike trains of V1 neurons are generated as inhomogeneous Poisson process with instantaneous rate

$$r_{i}\left(t\right)={\left[\iint F_{i}^{\left(k\right)}\left(x,y\right)\cdot {\tilde{m}}_{k}(x,y,t)\text{dxdy}\right]}_{+}$$ (5)

where ${\tilde{m}}_{k}\left(x,y,t\right)$ is the pixel value of image k (k=1,2) defined below (Eq. 7) and ${\left[x\right]}_{+}=\left\{\begin{array}{c} x,x\geq 0\\ 0,x<0 \end{array}\right.$ denotes half rectification. In the presence of image k, the average rate of $V1_{k}$ neurons was 10 Hz. In the absence of image k, $V1_{k}$ neurons had a spontaneous rate of 5 Hz.

Two Gabor images of orthogonal orientations are presented to the V1 neurons, either individually or simultaneously. Each image has 25 by 25 pixels and is defined as

$$\begin{array}{c} m_{k}\left(x,y\right)=c_{k}\text{exp}\left\{-\frac{1}{2\sigma^{2}}\left[{\left(x-x_{k}\right)}^{2}+{\left(y-y_{k}\right)}^{2}\right]\right\}\\ \text{cos}\left\{\frac{2\pi }{\lambda }\left[\left(x-x_{k}\right)\text{cos}\theta_{k}+\left(y-y_{k}\right)\text{sin}\theta_{k}\right]\right\} \end{array}$$ (6)

where $c_{k}$ is the contrast of image k, and σ and λ are the same as the Gabor filters of the V1 neurons (Eq. 3). Each pixel is corrupted by independent additive noise as

$${\tilde{m}}_{k}\left(x,y,t\right)=m_{k}\left(x,y\right)+\xi_{k}\left(x,y,t\right),k=1,2$$ (7)

where $\xi_{k}\left(x,y,t\right)$ is modeled as Ornstein-Uhlenbeck process

$$\tau_{n}d\xi_{k}=-\xi_{k}\mathrm{dt}+\sigma_{n}\mathrm{dW}$$ (8)

with $\tau_{n}=40$ ms, $\sigma_{n}=3.5$, and dW being a Wiener process.

#### V4/MT model neurons

V4/MT layer is a recurrently coupled network of excitatory (α=e) and inhibitory (α=i) neurons. The neuronal and synaptic parameters are the same as in our previous model (*20*). The preferred orientation of each neuron was given by the superimposition with a pinwheel map independently generated with the same method as that of V1 neurons (Eq. 4). Each neuron is modeled as an EIF neuron whose membrane potential is described by

$$C_{m}\frac{dV_{j}^{\alpha }}{\mathrm{dt}}=-g_{L}\left(V_{j}^{\alpha }-E_{L}\right)+g_{L}\Delta_{T}e^{\left(V_{j}^{\alpha }-V_{T}\right)/\Delta_{T}}+I_{j}^{\alpha }\left(t\right)$$ (9)

A spike is generated each time $V_{j}^{\alpha }\left(t\right)$ exceeds a threshold, *V*_th_. Then, the neuron’s membrane potential is held for a refractory period, $\tau_{\text{ref}}$, after which it is reset to a fixed value $V_{\text{re}}$. Neuron parameters for excitatory neurons are τ_m_ = *C*_m_/g_L_ = 15 ms, *E*_L_ = −60 mV, *V*_T_ = −50 mV, *V*_th_ = −10 mV, $\Delta_{T}=2$ mV, $V_{\text{re}}=-65$ mV, and $\tau_{\text{ref}}=1.5$ ms. Inhibitory neurons are the same except $\tau_{m}=10$ ms, $\Delta_{T}=0.5$ mV, and $\tau_{\text{ref}}=0.5$ ms. The total current to the jth neuron is

$$\frac{I_{j}^{\alpha }\left(t\right)}{C_{m}}=\sum_{k=1}^{N_{F}}\frac{J_{\mathrm{jk}}^{\alpha F}}{\sqrt{N}}\sum_{n}\eta_{F}\left(t-t_{n}^{F,k}\right)+\sum_{\beta =e,i}\sum_{k=1}^{N_{\beta }}\frac{J_{\mathrm{jk}}^{\alpha \beta }}{\sqrt{N}}\sum_{n}\eta_{\beta }\left(t-t_{n}^{\beta ,k}\right)$$ (10)

where $N=N_{e}+N_{i}$ is the total number of the network population. The postsynaptic current is given by

$$\eta_{\beta }\left(t\right)=\frac{1}{\tau_{\beta d}-\tau_{\beta r}}\left\{\begin{array}{cc} e^{-t/\tau_{\beta d}}-e^{-t/\tau_{\beta r}}, & t\geq 0\\ 0, & t<0 \end{array}\right.$$ (11)

where $\tau_{\text{er}}=1$ ms and $\tau_{\text{ed}}=5$ ms for excitatory synapses and $\tau_{\text{ir}}=1$ ms and $\tau_{\text{id}}=8$ ms for inhibitory synapses. The feedforward synapses from V1 neurons to V4/MT neurons have a fast and a slow component

$$\eta_{F}\left(t\right)=p_{f}\eta_{e}\left(t\right)+p_{s}\eta_{s}\left(t\right)$$ (12)

with $p_{f}=0.2$ and $p_{s}=0.8$. $\eta_{s}\left(t\right)$ has the same form as Eq. 11 with a rise time constant $\tau_{r}^{s}=2$ ms and a decay time constant $\tau_{d}^{s}=100$ ms.

#### Network connections

There are two types of feedforward and recurrent excitatory connections projecting to the excitatory V4/MT neurons. Eighty-five percent of connections are generated according to connection probability that depends only on the physical distance between neurons (Eq. 13). The remaining 15% of excitatory connections are randomly chosen from similarly tuned neurons and do not depend on space. Neuron i and j with preferred orientations of $\theta_{i}$ and $\theta_{j}$, respectively, are similarly tuned if $\text{cos}\left(\theta_{i}-\theta_{j}\right)\geq 0.6$. The probability of inhibitory projections and the projections to the inhibitory neurons only depend on distance (Eq. 13) and not on tuning similarity.

The distance-dependent connections are sampled according to probability function, $p_{\alpha \beta }\left(x_{1},x_{2}\right)$, between a neuron from population β at location $x_{1}=\left(x_{1},y_{1}\right)$ to a neuron from population α at location $x_{2}=\left(x_{2},y_{2}\right)$, α,β= {e, i}.

$$p_{\alpha \beta }\left(x_{1},x_{2}\right)={\bar{p}}_{\alpha \beta }g\left(x_{1}-x_{2},y_{1}-y_{2};\sigma_{\beta }\right)$$ (13)

Here, ${\bar{p}}_{\alpha \beta }$ is the mean connection probability, and

$$g\left(x,y;\sigma \right)=\frac{1}{2\pi \sigma^{2}}\left[\sum_{k=-\infty }^{\infty }e^{-{\left(x+2k\right)}^{2}/\left(2\sigma^{2}\right)}\right]\left[\sum_{k=-\infty }^{\infty }e^{-{\left(y+k\right)}^{2}/\left(2\sigma^{2}\right)}\right]$$ (14)

is a wrapped Gaussian distribution periodic on the domain Γ. The excitatory and inhibitory recurrent connection widths of the V4/MT layer were $\sigma_{e}=\sigma_{i}=0.2$, and the feedforward connection width from the V1 layer to the V4/MT layer was $\sigma_{\text{ffwd}}=0.1$. A presynaptic neuron was allowed to make more than one synaptic connection to a single postsynaptic neuron.

The long-range excitatory connections are sampled between similarly tuned neurons, i.e., $\text{cos}\left(\theta_{i}-\theta_{j}\right)\geq 0.6$, where $\theta_{i}$ and $\theta_{j}$ are the preferred orientations of neuron i and j.

The recurrent synaptic weights within the V4/MT layer were $J_{\text{ee}}=80$ mV, $J_{\text{ei}}=-240$ mV, $J_{\text{ie}}=40$ mV, and $J_{\text{ii}}=-300$ mV. Note that each synaptic weight was scaled by $1/\sqrt{N}$ (Eq. 10). The mean connection probabilities were ${\bar{p}}_{\text{ee}}=0.01$, ${\bar{p}}_{\text{ei}}=0.04$, ${\bar{p}}_{\text{ie}}=0.03$, and ${\bar{p}}_{\text{ii}}=0.04$. The out-degrees were $K_{\text{ee}}^{\text{out}}=200$, $K_{\text{ei}}^{\text{out}}=800$, $K_{\text{ie}}^{\text{out}}=150$, and $K_{\text{ii}}^{\text{out}}=200$. The feedforward connection strengths from V1 layer to V4/MT layer were $J_{\text{eF}}=160\;\text{mV}$ and $J_{\text{iF}}=140$ mV, with probabilities ${\bar{p}}_{\text{eF}}=0.05$ and ${\bar{p}}_{\text{iF}}=0.05$ (out-degrees $K_{\text{eF}}^{\text{out}}=1000$ and $K_{\text{iF}}^{\text{out}}=250$).

#### Network with matched in-degrees

In the network with matched-in-degrees (Fig. 6), the number of presynaptic neurons (in-degrees) that project to each V4/MT neuron was matched to be the same across each population for each type of connections (V4/MT excitatory, V4/MT inhibitory, or V1 excitatory), while the out-degrees were allowed to vary. All other parameters including synaptic weights and connection probabilities were the same as the default network.

#### Simulation

All simulations were performed on the CNBC Cluster in the University of Pittsburgh. All simulations were written in a combination of C and Matlab (Matlab R 2021b, MathWorks). The differential equations of the neuron model were solved using forward Euler method with time step of 0.05 ms.

### Datasets and analysis

Neuronal activity was collected from four adult male rhesus monkeys (*Macaca mulatta*; monkeys BR, JD, ST, and SY) as they were passively fixating at superimposed orthogonal drifting gratings at a range of contrasts [details are described in (*12*)]. All animal procedures were approved by the Institutional Animal Care and Use Committees of the University of Pittsburgh and Carnegie Mellon University.

MT data were collected with 24-channel V-Probes and 24-channel linear microarrays in area MT of two monkeys (Fig. 2B). There were a total of 2133 visual stimulus trials for 769 units and 10,600 pairs from 28 recording sessions. V4 data were collected with a pair of 6 by 8 microelectrode arrays implanted in area V4 of two monkeys (Fig. 2C). In one monkey, both arrays were in V4 in the right hemisphere. In the other monkey, arrays were implanted bilaterally in area V4. There were a total of 2160 visual stimulus trials for 1276 units and 39,719 pairs from 21 recording sessions. V1 data were collected with a 10 by 10 microelectrode array implanted in area V1 of two monkeys (fig. S7). There were a total of 1467 visual stimulus trials for 2124 units and 97,169 pairs from 23 recording sessions.

In our analysis, we included units if their response to 0% contrast stimuli was significantly different from the average response to stimuli with at least 50% contrast (t test, P<0.01). Pairs of units that came from the same electrode were excluded for correlation analysis. For each unit recorded in each stimulus condition, spike counts were calculated from 30 to 230 ms for V1 units and from 50 to 250 ms for V4 and MT units after stimulus onset to allow for latency in response. Each stimulus was presented for 200 ms. We quantified spike count correlations as the Pearson’s correlation coefficient between spike count responses to repeated presentations of the same stimulus. This measure is extremely sensitive to outliers, so we did not analyze trials for which the response of either unit was more than three SDs away from its mean [following the convention of (*81*)].

To compute the normalization index of recorded units, we included the mean spike counts in response to stimulus conditions where the contrast of one stimulus was 50% and the other was 0% and where the contrast of both stimuli was 50%. The normalization index was computed using Eq. 15 for each combination of orthogonal drifting gratings and then averaged across all combinations within a session. To compute the selectivity of recorded units, we included the mean spike counts in response to stimulus conditions where the contrast of one stimulus was 50% and the other was 0%. Tuning similarity was quantified as the Pearson’s correlation coefficient between mean spike count responses to each stimulus direction presented alone, with 50% contrast and the contrast of the other direction equal to 0%. The spike count correlation of a pair of units in one session was averaged across the stimulus conditions used to compute the normalization index. In Fig. 2 (B and C) and fig. S7, the spike count correlation was averaged across unit pairs from all recording sessions.

### Statistical methods

#### Normalization index

The normalization index of a neuron is defined as the sum of a neuron’s firing rate to each one of the two stimuli when presented alone divided by its firing rate when both stimuli are presented together (*12*)

$$\text{Norm index}=\left({\text{FR}}_{\text{stim}1}+{\text{FR}}_{\text{stim}2}\right)/{\text{FR}}_{\text{both}}$$ (15)

A normalization larger than 1 indicates sublinear summation.

The current normalization index is defined similarly

$$\text{Current}\;\text{norm}\;\text{index}=\left(I_{\text{stim}1}+I_{\text{stim}2}\right)/I_{\text{both}}$$ (16)

where I is the average recurrent excitatory, recurrent inhibitory, or feedforward excitatory current that a neuron receives.

#### Spike count correlation and current covariance

We computed the spike count correlation of V4/MT model neurons when both images of orthogonal orientations were presented together. Spike counts were computed using a sliding window of 200 ms with 50-ms step size, and the Pearson correlation coefficients were computed between pairs of neurons. In Fig. 2A, there were 10 simulations, and each simulation was 20 s long. The first 1 s of each simulation was excluded from the correlation analysis to avoid transient effects. Neurons whose average firing rates were within one SD from the mean population rates in all of the three stimulus conditions (image 1 alone, image 2 alone, or both images presented together) were included. In total, 8663 excitatory V4/MT neurons were sampled to compute spike count correlations.

To compute current covariance (Fig. 3, D and E), each type of synaptic currents (feedforward excitation, recurrent excitation, and recurrent inhibition) to each neuron was recorded every 10 ms. The excitatory current combines both feedforward and recurrent excitation. In total, 4163 neurons were sampled to compute current covariance. The simulation was 20 s long.

#### Tuning similarity

Tuning similarity between a pair of neurons is defined as the Pearson correlation between their tuning curves of orientation

$$\text{Tuning similarity}=\text{corr}\left[f_{i}\left(\theta \right),f_{j}\left(\theta \right)\right]$$ (17)

where $f_{i}$ and $f_{j}$ are the tuning curves of neuron i and neuron j, respectively. Tuning curves were computed with Gabor images presented at location 1. The orientations of the presented Gabor image were between 0° degree and 180° with a uniform spacing of 10°.

#### Selectivity

Selectivity measures how selective a neuron is to stimulus 1 compared to stimulus 2

$$\text{Selectivity}=\frac{{\text{FR}}_{\text{stim}1}-{\text{FR}}_{\text{stim}2}}{{\text{FR}}_{\text{stim}1}+{\text{FR}}_{\text{stim}2}}$$ (18)

Selectivity takes a value between −1 and 1. The larger the absolute value of selectivity is, the more selective the neuron is to its preferred stimulus. A selectivity equaling 0 means that there is no preference of the neuron between the two stimuli.

#### Linear Fisher information

To compute the linear Fisher information, stimulus 2 was presented during 200-ms intervals (ON) interleaved with 300-ms OFF intervals, during which the spike trains of V1_2_ neurons were independent Poisson process with rate of 5 Hz. Meanwhile stimulus 1 was present throughout a simulation. Spike counts of V4/MT excitatory neurons during the ON intervals were used to compute the linear Fisher information. Each simulation was 20 s long. The first spike count in each simulation was excluded. The connectivity matrices were fixed for all simulations, and the initial state of each neuron’s membrane potential was randomized in each simulation.

For the linear Fisher information of contrast, the contrast of stimulus 2 during ON intervals was randomly chosen from $c_{1}=c+\delta c/2$ and $c_{2}=c-\delta c/2$, where c=0.5 and δc=0.01. For the linear Fisher information of orientation, the orientation of stimulus 2 during ON intervals was randomly chosen from $\theta_{1}=\theta +\delta \theta /2$ and $\theta_{2}=\theta -\delta \theta /2$, where θ=0.5π rad and δθ=0.02 rad. The linear Fisher information of V4/MT neurons was computed using a bias-corrected estimate (*44*)

$${\hat{I}}_{\mathrm{bc}}=\frac{{\left(f_{2}-f_{1}\right)}^{⊤}}{\delta x}{\left(\frac{Q_{1}+Q_{2}}{2}\right)}^{-1}\frac{\left(f_{2}-f_{1}\right)}{\delta x}\frac{2N_{\text{tr}}-N-3}{2N_{\text{tr}}-2}-\frac{2N}{N_{\text{tr}}\delta x^{2}}$$ (19)

where δx=δc or δθ, and $f_{i}$ and $Q_{i}$ are the empirical mean and covariance, respectively, for $c_{i}$ or $\theta_{i}$. $N_{\text{tr}}$ is the number of trials for each $c_{i}$ or $\theta_{i}$.

We used the fitting algorithm proposed by (*82*) to estimate the asymptotic value of the linear Fisher information, $I_{\infty }$, at the limit of N→∞, where N is the number of sampled neurons (Fig. 6, B and C). Briefly, the theory of information-limiting correlations (*83*) shows that the linear Fisher information, $I_{N}$, in a population of N neurons can be decomposed into a limiting component, $I_{\infty }$, and a nonlimiting component $I_{0}\left(N\right)$

$$I_{N}=\frac{1}{\frac{1}{I_{0}}+\frac{1}{I_{\infty }}}$$ (20)

where we assume that the nonlimiting component increases linearly with N, i.e., $I_{0}=\text{aN}$. Hence, Eq. 20 can be rewritten as

$$1/I_{N}=\left(1/a\right)\left(1/N\right)+1/I_{\infty }$$ (21)

which shows that $1/I_{N}$ scales linearly with 1/N with $1/I_{\infty }$ as the intercept. Hence, we do a linear fit of $1/I_{N}$ versus 1/N, with N varying from 8 to 12,000 and estimate $1/I_{\infty }$.

The linear Fisher information from V1 neurons can be estimated analytically as (*80*)

$$I_{F}^{\text{in}}\left(s\right)={\frac{\partial f}{\partial s}}^{⊤}\Sigma^{-1}\left(s\right)\frac{\partial f}{\partial s}$$

where s=c or θ, with

$$\frac{\partial f}{\partial s}={\mathrm{TF}}_{i}\cdot \frac{\partial m\left(c,\theta \right)}{\partial s}$$

$$\Sigma_{\mathrm{ij}}\left(s\right)=F_{i}\cdot F_{j}\text{Var}\left(\xi^{T}\right)+\delta_{\mathrm{ij}}{\mathrm{TF}}_{i}\cdot m\left(c,\theta \right)$$

where $\xi^{T}\left(t\right)=\int_{t}^{t+T}\xi \left(u\right)\mathrm{du}$, T is the time window for spike counts, and $\delta_{\mathrm{ij}}$ is a Kronecker delta, which is 1 if i=j, and 0 otherwise. We can calculate the variance of the integrated noise over time window T as $\text{Var}\left(\xi^{T}\right)=\sigma_{n}^{2}\left\{T-\tau_{n}\left[1-\text{exp}\left(-T/\tau_{n}\right)\right]\right\}$.

In Fig. 5, a number of neurons were randomly sampled from the model V4/MT excitatory neurons whose normalization indices were within a specified range. The number of sampled neurons, N, was logarithmically spaced between 1 and 8103. For N<545 neurons, the number of samplings for each N decreased from 200 to 14 proportionally with log(N), rounded to the nearest integer. For N≥545 neurons, there were five samplings for each N. The linear Fisher information of the sampled group of neurons was divided by the average number of spikes from the sampled group of neurons during the time window used for calculating the Fisher information (T=200 ms). In Fig. 6 (B and C), the number of neurons was *N* = 8, 16, 31, 62, 125, 250, 500, 1000, 2000, 4000, 8000, and 12,000. There were 20 samples of neurons for each *N*. Neurons with firing rates less than 1 Hz were excluded. There were 304,000 spike counts in total for $c_{1}$ and $c_{2}$ or for $\theta_{1}$ and $\theta_{2}$ conditions.

#### Manifold capacity analysis

We quantified the manifold capacity of neural representations using a statistical mechanical framework on the basis of replica mean field theory, following the formalism introduced in (*45*, *46*). In the neural activity space, let N denote the number of neurons and P the number of object manifolds (i.e., distinct natural images). The manifold capacity α is defined as the critical load ratio α=P/N, which represents the maximal number of linearly separable object manifolds per neuron. Our implementation follows the procedure described in (*46*), where manifold anchor points and their geometric properties are derived from the neural population responses. To understand the factors that influence manifold capacity, we computed two key geometric descriptors of each manifold—manifold dimension, $D_{M}$, and manifold radius, $R_{M}$—using the method from (*46*).

The manifold dimension ($D_{M}$**)** measures the spread of the manifold anchor points along the different manifold axes. It is defined as

$$D_{M}={\langle \vec{t}\cdot \hat{s}\rangle }_{\overset{\to }{t},t_{0}}$$ (22)

where $\hat{s}$ is a unit vector in the direction of anchor points $\tilde{s}$, and ${\langle \ldots \rangle }_{\overset{\to }{t},t_{0}}$ is an average over random D- and one-dimensional vectors $\vec{t}$ and $t_{0}$ whose components are independent and normally distributed $t_{i}∼N\left(0,1\right)$. The anchor points, $\tilde{s}$, uniquely given by each $\vec{t}$ and $t_{0}$, represent a weighted sum of support vectors contributing to the linearly separating solution. D is the dimension of the coordinates in which each manifold is embedded. The manifold dimension estimates the average embedding dimension of the manifolds contributing to classifications.

The manifold radius ($R_{M}$**)** measures the average distance between the manifold center and the anchor points and is defined as

$$R_{M}=\sqrt{{\langle {\tilde{s}}^{2}\rangle }_{\overset{\to }{t},t_{0}}}$$ (23)

$R_{M}$ is the size relative to the norm of the manifold center.

These two quantities, $D_{M}$ and $R_{M}$, together characterize the shape and size of the manifolds in the neural representation space. According to the mean-field theory of manifold capacity, manifolds with lower dimension and smaller radius are more likely to be linearly separable and result in higher manifold capacity (*45*).

To simulate the response of the network to natural images, we selected a set of 50 photographs from the ImageNet database. Each image was converted to gray scale and downsampled to 114 by 64 pixels. For each image input, the mean firing rate of each V1 model neuron was obtained by convolving its Gabor receptive-field filter, $F_{i}^{\left(k\right)}\left(x,y\right)$ (Eq. 3), with the corresponding 15 by 15 pixel patch of the image. Receptive fields of V1 neurons tiled the visual space with a stride of one pixel; consequently, the receptive fields of neighboring neurons overlapped by 14 pixels in both directions. V1 firing rates were normalized such that the mean population firing rate was 10 Hz.

To quantify how the manifold capacity scales with population size, excitatory V4/MT neurons were randomly subsampled at four sizes: N=200,400,800,1600, and 3200. For every N, we generated 20 independent subsamples. For each N, we simulated M=0.2N trials per image; each trial consisted of the spike counts from a 100-ms time window. To compute the manifold capacity of the set of image-evoked population responses, we sampled $n_{t}=200$ random direction vectors $\vec{t}$ and used a margin κ=0 for linear separability (*46*).

## Supplementary Materials

### This PDF file includes:

Supplementary Note 1 Figs. S1 to S17 References
