# Supplementary material for: Neuronal heterogeneity of normalization strength in a circuit model
Source: Sci Adv. 2026 Jan 1;12(1):eadv9396. doi: 10.1126/sciadv.adv9396 (PMC12757059; doi:10.1126/sciadv.adv9396)
Supplement: Supplementary file 1 — Supplementary Note 1 Figs. S1 to S17 References [file sciadv.adv9396_sm.pdf]

Science Advances
AAAS

Supplementary Materials for

Neuronal heterogeneity of normalization strength in a circuit model

Deying Song *et al.*

Corresponding author: Chengcheng Huang, huangc@pitt.edu

Sci. Adv. **12**, eadv9396 (2026)
DOI: 10.1126/sciadv.adv9396

This PDF file includes:

Supplementary Note 1
Figs. S1 to S17
References

Supplementary Note 1: Impacts of transfer function on normalization heterogeneity

Feedforward model

We first consider a simple scenario where the rate of a neuron only depends on its feedforward input and its input-output transfer function, i.e. $r_k = f(I_k)$, where $k = \{1, 2, 1+2\}$ indicates the stimulus condition (Stimulus 1 alone, Stimulus 2 alone or Stimulus 1 and Stimulus 2 together). We assume that the inputs are linear with respect to stimulus summation and analyze how the non-linearity of the transfer function, $f(I)$, can shape the normalization index of firing rates.

Specifically, we assume $I_{1+2} = (I_1 + I_2)/1.5 + I_0$, which is the case for the feedforward currents from our network model (fig. S11Aa magenta), and a good approximation for the total current each neuron receives (recurrent and feedforward) in our model (fig. S11Aa cyan). We assume that the transfer function is a rectified power-law function, i.e.

$$f(I) = \begin{cases} a \cdot [I - \theta]^n, I > \theta \\ 0, I \leq \theta \end{cases} . \tag{S1}$$

The transfer function of the neurons in our network model can be approximated by a power-law of $n \approx 2.45$ at low rates ($\leq$ 20 Hz) and a power-law of $n = 0.87$ at high rates (> 20 Hz) (fig. S11Ab). We consider the case when rates are positive at all three stimulus conditions, i.e. $r_1 > 0$, $r_2 > 0$ and $r_{1+2} > 0$. If the rate to one stimulus is zero, e.g. $r_1 = 0$, then I_1 can be arbitrarily negative, resulting in a small r_{1+2} and a large normalization index. This scenario is discussed in (*55*) as a potential mechanism to produce large normalization in balanced networks.

The normalization index of this feedforward model can be calculated as

$$NI = \frac{r_1 + r_2}{r_{1+2}} = \frac{a(I_1 - \theta)^n + a(I_2 - \theta)^n}{a(I_{1+2} - \theta)^n} \tag{S2}$$

$$= \frac{(I_1 - \theta)^n + (I_2 - \theta)^n}{(I_1 + I_2)/1.5 + I_0 - \theta)^n} \tag{S3}$$

$$= \frac{x^n + y^n}{((x + y)/1.5 + (I_0 + \theta/3))^n}, \tag{S4}$$

with a change of variable $x = I_1 - \theta > 0$ and $y = I_2 - \theta > 0$. If $n \geq 1$ and $I_0 + \theta/3 \geq 0$, we have

$$NI \leq \frac{x^n + y^n}{(x/1.5)^n + (y/1.5)^n + (I_0 + \theta/3)^n} \tag{S5}$$

$$\leq \frac{x^n + y^n}{(x/1.5)^n + (y/1.5)^n} \tag{S6}$$

$$\leq (1.5)^n. \tag{S7}$$

If $I_0 + \theta/3 < 0$, then the denominator of Eq. S4 can be close to zero with small x and y, resulting in a large normalization index.

We demonstrate the dependence of the normalization index on the firing rates r_1 and r_2 for different types of transfer functions (fig. S11, B to D). If the transfer function is superlinear, i.e. $n > 1$, and $I_0 + \theta/3 > 0$, the normalization index is larger than 1.5 when only one of r_1 and r_2 is small, and is smaller than 1.5 otherwise (fig. S11, Ba and Bb). When I_0 is more negative ($I_0 + \theta/3 < 0$), the model can achieve a much larger normalization index in regions where r_1 or r_2 is small (fig. S11Bc). If the transfer function is a rectified-linear function, the normalization index is close to 1.5 except for when both r_1 and r_2 are very small and I_0 is sufficiently negative (fig. S11, Ca to Cc). If the transfer function is sublinear, the normalization index is large when r_1 and r_2 are similar and is small when one of r_1 and r_2 is small (fig. S11, Da to Dc), opposite to the patterns with a superlinear transfer function.

The dependence of the normalization index on the firing rates r_1 and r_2 in our spiking network model is similar to that in the feedforward model with a superlinear transfer function (fig. S11, Ac and Ad compared to Bb and Bc). For neurons that receive more excitatory input when both stimuli are presented (larger I_0), their normalization indices are large if they selectively respond to one of the two stimuli (r_1 large or r_2 large) (fig. S11Ac). For neurons that receive more inhibitory input when both stimuli are presented (more negative I_0), their normalization indices are generally larger, especially when their responses to one of the stimuli are low (fig. S11Ad). We observe similar result for the inhibitory neurons (fig. S12, Aa and Ab).

This analysis explains why stimulus overlap affects the distribution of normalization indices. When the two stimuli activate highly overlapped neuron populations, the firing rates of each neuron for the two stimulus conditions, r_1 and r_2, are highly correlated (fig. S12, Ba and Bb). Therefore, the network lacks neurons that respond selectively to stimulus 1 or stimulus 2, which are of strong normalization (fig. S11, Ac and Ad).

Recurrent model

Second, we investigate how transfer function shapes normalization heterogeneity in a recurrent network of rate units. Since the transfer function in our spiking network model is an emergent property of the recurrent network, it is not straightforward to manipulate the transfer function. Instead, we study this in a firing rate model where the transfer function is explicitly given and the connectivity matrix is the same as that in the spiking network model.

The recurrent model consists of rate units governed by the following dynamics:

$$\tau_\alpha \frac{dr_\alpha^j}{dt} = -r_\alpha^j + \phi_\alpha\left(\sum_{k=1}^{N_{\rm F}} \frac{J_{jk}^{\alpha \rm F}}{\sqrt{N}} \sum_n r_F^k + \sum_{\beta={\rm e,i}} \sum_{k=1}^{N_\beta} \frac{J_{jk}^{\alpha\beta}}{\sqrt{N}} \sum_n r_\beta^k\right), \tag{S8}$$

where τ_α is the time constant of neurons in population α and $\phi_\alpha(x)$ is the transfer function of population α, $\alpha = {\rm e,i}$. e, i, F represents the V4/MT excitatory, V4/MT inhibitory, and V1 populations, respectively. The recurrent and feedforward connectivity matrices ($J^{\alpha\beta}$ and $J^{\alpha F}$, respectively) are the same as those in the spiking neuron network. With the transfer functions fitted to those from the spiking network (fig. S17A), the rate model reproduces the mean firing rates of individual neurons in the spiking neuron model (fig. S17 B).

We compare three alternative transfer functions, each with the same threshold and dynamic range as those of the spiking network model, but with a different power-law exponent: n = 0.8 (sublinear), n = 1 (linear), and n = 2 (supralinear) (fig. S17 Aa and Ab). We find that the model with a sublinear transfer function exhibits greater heterogeneity in normalization index compared to models with a linear or a supralinear transfer function, and the spiking network model (fig. S17 C). These findings demonstrate that the shape of the transfer function plays a substantial role in shaping the distribution of normalization indices.

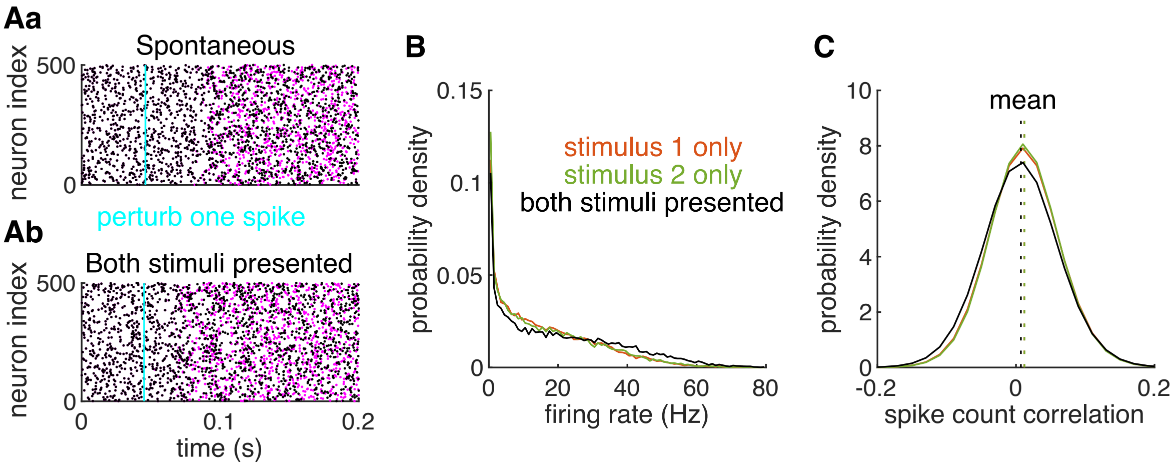

Figure S1: **Related to Fig. 1. The V4/MT network activity is stable and asynchronous in both spontaneous and evoked states.** (**Aa**) Spike raster of V4/MT neurons when both $V1_1$ and $V1_2$ neurons have homogeneous rates of 10 Hz. 500 neurons were randomly sampled from the V4/MT population. The magenta spike trains are from a trial with the same V1 input spike trains and initial conditions as the black spike trains, except that one spike from the V4/MT neurons was removed at the time point indicated by the cyan line. (**Ab**) Spike raster of V4/MT neurons when two images are simultaneously presented. (**B**) The firing rate distributions of V4/MT neurons when only stimulus 1 (red), only stimulus 2 (green) or both stimuli are presented (black). (**C**) The distributions of spike count correlations of V4/MT neuron pairs from the three stimulus conditions. Dashed lines indicate the average spike count correlations (0.012 when only stimulus 1 is presented, 0.012 when only stimulus 2 is presented, 0.007 when both stimuli are presented).

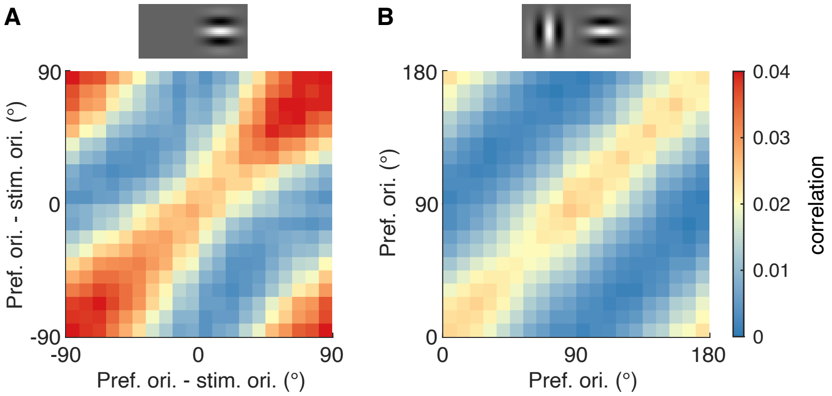

Figure S2: **Related to Fig. 1. The spike count correlations are higher between similarly tuned neurons and depend on stimulus orientation.** (**A**) When presented with one Gabor image, the spike count correlations are higher between similarly tuned neurons. Among neuron pairs with similar preferred orientations, the spike count correlations are higher between pairs with preferred orientations orthogonal to the stimulus orientation than those with similar preferred orientations. (**B**) When two Gabor images with orthogonal orientations are presented together, the spike count correlations are also higher between similarly tuned neurons.

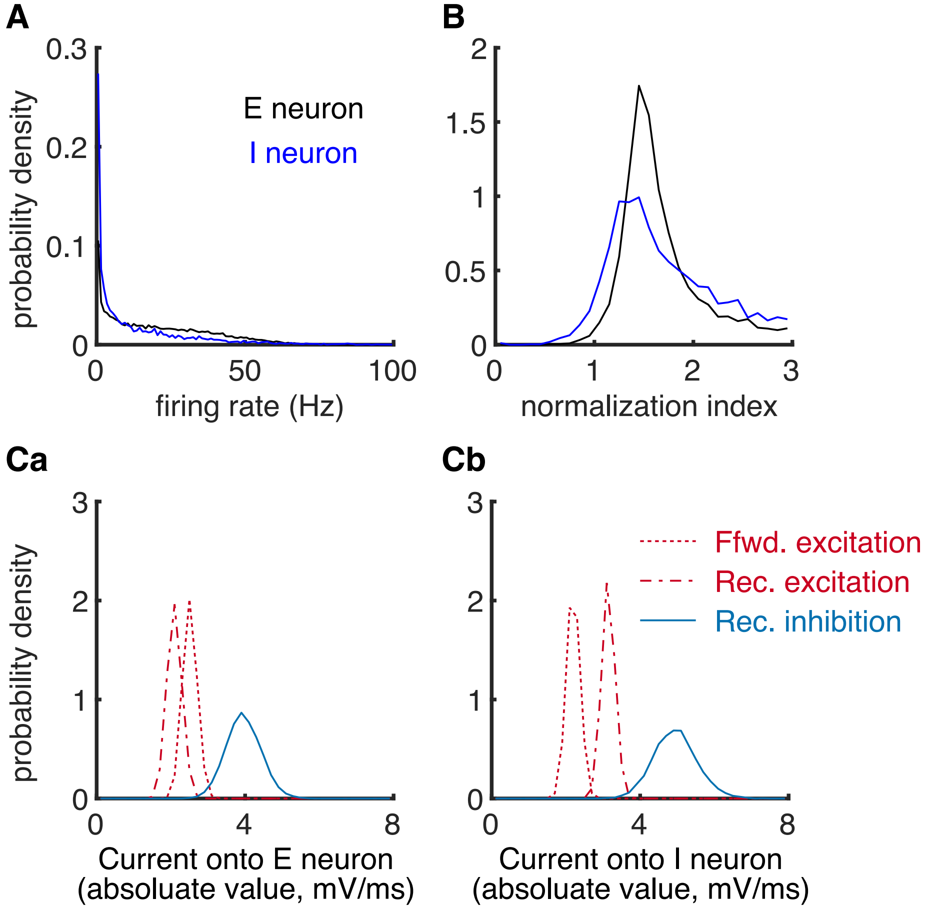

Figure S3: **Related to Fig. 3. There is more heterogeneity in both inhibitory activity and inhibitory input.** (**A**) The distribution of firing rates of inhibitory neurons is more skewed (heavy-tailed) than that of excitatory neurons. (**B**) The distribution of normalization indices of inhibitory neurons is wider than that of excitatory neurons. (**Ca**) Population distributions of mean recurrent excitatory, feedforward excitatory and recurrent inhibitory currents for excitatory neurons. The distribution of the inhibitory current is broader than that of feedforward excitation and recurrent excitation. (**Cb**) Same as **Ca** for the inhibitory neurons.

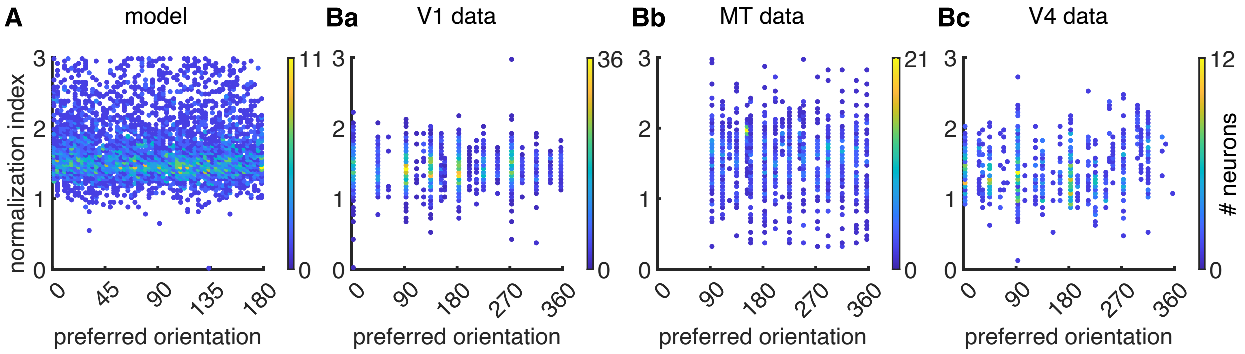

Figure S4: **Related to Fig. 2. The normalization index of a neuron is independent from its tuning preference in both the model and data.** (**A**) The normalization index and the tuning preference of model V4/MT neurons are statistically independent. We use permutation test to assess the statistical significance of the null hypothesis that the normalization index and tuning preference are independent. First, we calculate the mutual information between the normalization index and tuning preference of V4/MT neurons. Then, we shuffle the normalization index of neurons 2000 times and recalculate the mutual information for each permutation. Lastly, we compute the p-value as the proportion of permutations with higher mutual information than that of the original observation. There is no significant dependence between the normalization index and the tuning preference of model V4/MT neurons ($p = 0.31$). (**B**) The normalization index and the tuning preference of experimentally recorded neurons are statistically independent in V1 area (**Ba**, permutation test, $p > 0.05$ for all 23 recording sessions), MT area (**Bb**, $p > 0.05$ for 21 out of 28 recording sessions), and V4 area (**Bc**, $p > 0.05$ for 14 out of 21 recording sessions). Data in panels B are from (*12*).

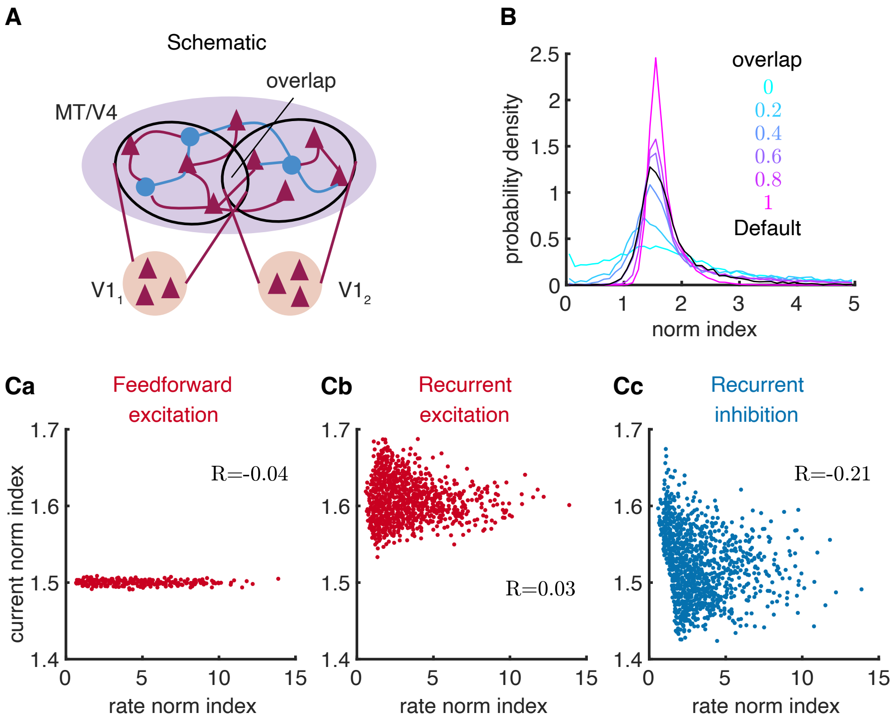

Figure S5: **Related to Figures 1 and 3. Neurons exhibit a range of normalization strength in networks with strong recurrent coupling and disordered connections.** (**A**) The schematic of the disordered network. The excitatory and inhibitory connections within V4/MT layer are random and with the same connection probability as those of the default model with spatial structure. The two V1 populations project randomly to the excitatory and inhibitory neurons in the V4/MT layer with the same connection probabilities as those in the default model. A fraction, α, of neurons in the V4/MT layer can receive input from both V1 populations, which represents the overlap of the feedforward projections from $V1_1$ and $V1_2$. The spike trains of V1 neurons are independent Poisson processes with fixed rates. In Stimulus 1 alone condition, $V1_1$ neurons have firing rate of 10Hz and $V1_2$ neurons have rate 5Hz. In Stimulus 2 alone condition, $V1_1$ neurons have firing rate of 5Hz and $V1_2$ neurons have rate of 10Hz. When both stimuli are presented, both $V1_1$ and $V1_2$ neurons fire at 10 Hz. (**B**) The distribution of normalization indexes is broader in networks with smaller overlap, α. The distribution of normalization indexes from the default network (black; same as that in fig. 1C) is similar to that of the random network model with $\alpha = 0.6$. The normalization index of neurons in the random network model is calculated in the same way as in the default model, i.e. norm index $= (FR_{stim1} + FR_{stim2})/FR_{both}$. (**C**) Relationship between current and rate normalization indexes in a random network model with $\alpha = 0.6$. Only the normalization index of recurrent inhibitory current has a strong correlation with that of the firing rate. The range of the normalization index of feedforward excitatory current is very small in the random network model, since there is no spatial or tuning dependent connections. ((**Ca**), Pearson correlation, $r = -0.04$, $p < 0.01$, $N = 7,542$, (**Cb**), $r = 0.03$, $p < 0.01$, (**Cc**), $r = -0.21$, $p < 10^{-4}$.)

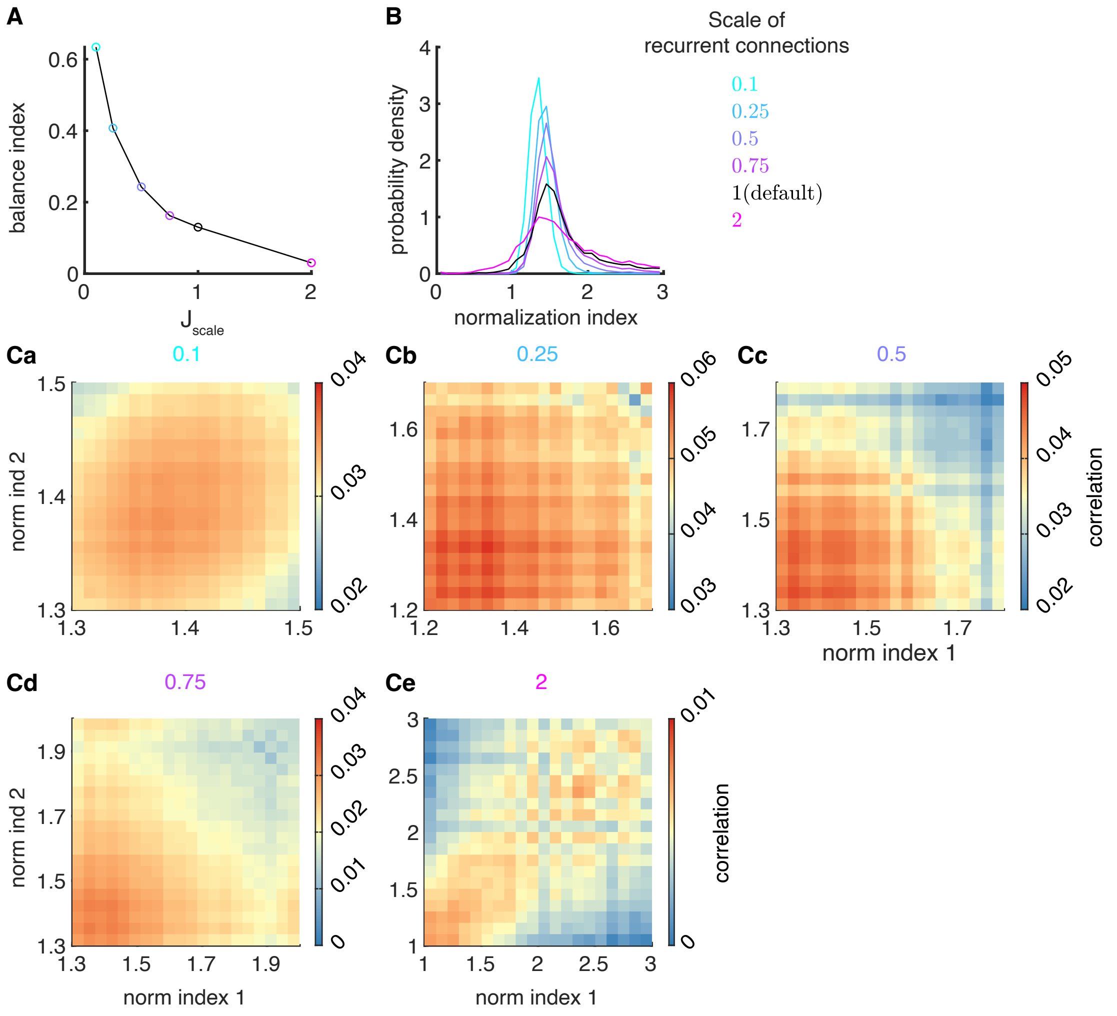

Figure S6: **Related to Fig. 2. Networks with weak recurrent coupling produce homogeneous normalization strength and weak dependence of spike count correlations on normalization.** The strengths of all recurrent connections were scaled by a common factor J_{scale}, $J_{\text{scale}} = 0.1, 0.25, 0.5, 0.75, 2$. (**A**) The balance index decreases with increasing recurrent connection strength. The balance index is defined as $\frac{|\mu|}{\mu_E+\mu_X}$, where μ is net mean current, μ_E and μ_X are the mean recurrent and external excitation, respectively (*84*). A smaller balanced index means tighter balance. (**B**) Stronger recurrent connection strength leads to a broader distribution of normalization indices. (**Ca-Ce**) The dependence of spike count correlations on normalization strength in networks with different coupling strengths. The spike count correlations weakly depend on normalization indices when the recurrent coupling is weak (**Ca-Cb**). The dependence of spike count correlations on normalization indices is consistent with data when the recurrent coupling is strong (**Cc-Cd**). The spike count correlations become smaller and have weaker dependence on normalization indices when the network is in a tightly balanced regime (**Ce**). In the network with weak recurrent coupling ($J_{\text{scale}} < 1$), independent white noise currents with mean 0 and standard deviation 6.8 mV/ms were applied to every excitatory and inhibitory V4/MT neuron such that the neurons' f-I curve was the same as that in the default network. The strengths of feedforward projections were scaled by a factor $J_{\text{scale,ffwd}}$ to keep the mean firing rate of V4/MT neurons the same as that in the default network (for $J_{\text{scale}} = 0.1$, $J_{\text{scale,ffwd}} = 0.34$; for $J_{\text{scale}} = 0.25$, $J_{\text{scale,ffwd}} = 0.45$; for $J_{\text{scale}} = 0.5$, $J_{\text{scale,ffwd}} = 0.63$; for $J_{\text{scale}} = 0.75$, $J_{\text{scale,ffwd}} = 0.83$; for $J_{\text{scale}} = 2$, $J_{\text{scale,ffwd}} = 2$). The default network was the same as that used in Fig. 1 and 2.

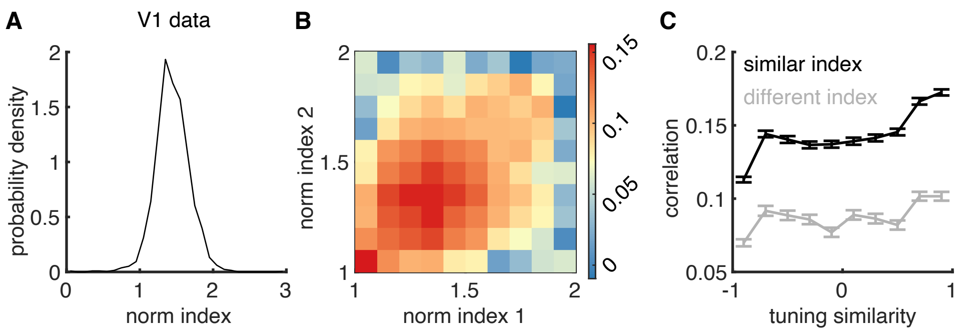

Figure S7: **Related to Figures 1 and 2. Heterogeneity of normalization index and normalization-related modulation of spike count correlations are also observed in experimental data recorded from V1 area.** (**A**) The recorded V1 neurons exhibit a similar range of normalization indexes as V4 and MT neurons (fig. 1D). (**B**) Spike count correlations between recorded V1 neurons depend on their normalization indexes. Same format as fig. 2, Aa,Ba and Ca). (**C**) Across all levels of tuning similarity, the spike count correlations between recorded V1 neurons with similar normalization indexes are consistently larger than those of neurons with distinct normalization indexes. Error bars represent the SEM. Same format as fig. 2, Ad,Bd, and Cd). Data from (*12*).

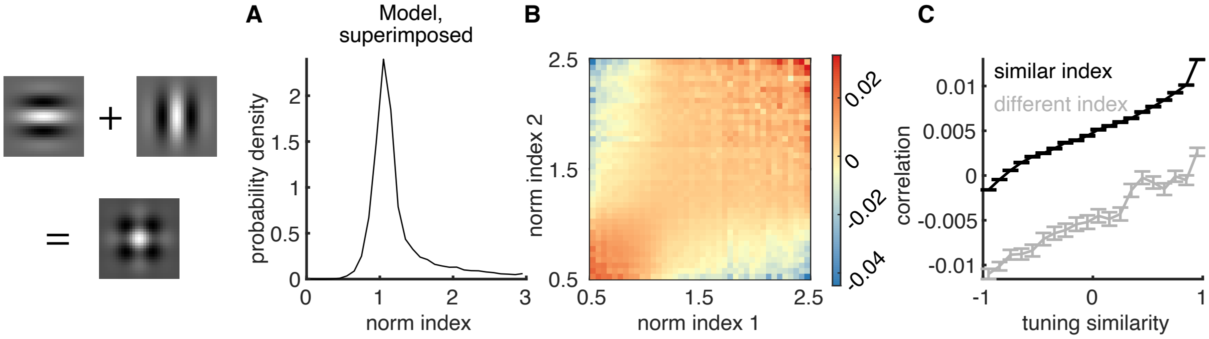

Figure S8: **Related to Figures 1 and 2. Heterogeneity in normalization index and normalization-related modulation of spike count correlations in the model in response to two superimposed Gabor images.** (**A**) Distribution of normalization indexes (Eq. 15 where FR_{both} was the firing rate when two superimposed Gabor images were presented. (**B**) Spike count correlations as a function of the normalization indexes of the two neurons in the pair. (**C**) Neurons with similar normalization indexes have higher noise correlations than those with different normalization indexes. Error bars represent the SEM. This relationship is consistent across neuron pairs with various tuning similarities. Model results with superimposed Gabor images are qualitatively the same as those with two separate Gabor images (Fig. 1C, 2Aa, and 2Ad).

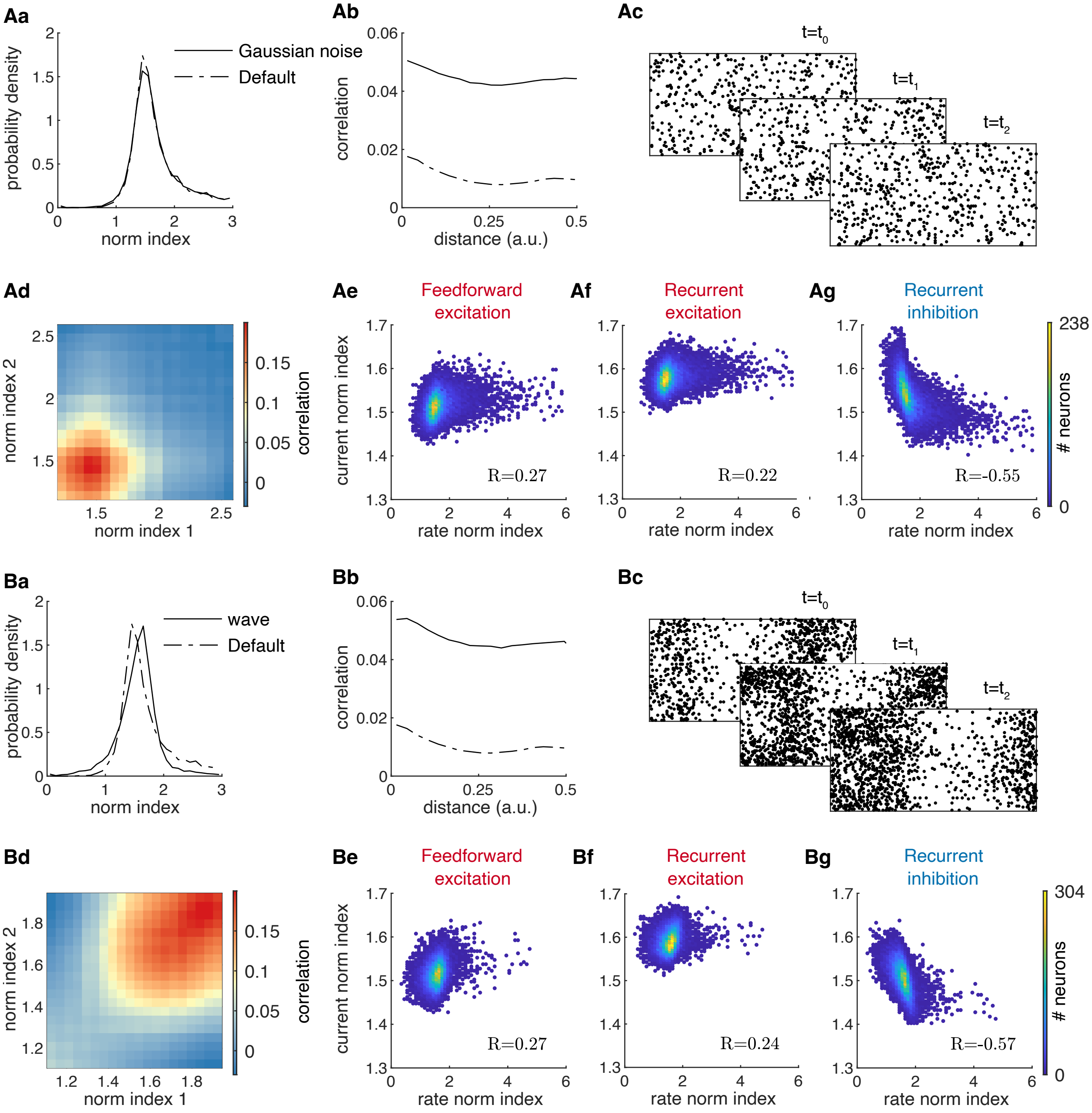
Aa
Gaussian noise
Default
probability density
norm index
Ab
correlation
distance (a.u.)
Ac
t=t0
t=t1
t=t2
Ad
norm index 2
norm index 1
correlation
Ae
Feedforward excitation
current norm index
rate norm index
R=0.27
Af
Recurrent excitation
rate norm index
R=0.22
Ag
Recurrent inhibition
rate norm index
R=-0.55
238
neurons
Ba
wave
Default
probability density
norm index
Bb
correlation
distance (a.u.)
Bc
t=t0
t=t1
t=t2
Bd
norm index 2
norm index 1
correlation
Be
Feedforward excitation
current norm index
rate norm index
R=0.27
Bf
Recurrent excitation
rate norm index
R=0.24
Bg
Recurrent inhibition
rate norm index
R=-0.57
304
neurons

Figure S9: **Related to Fig. 2 and Fig. 3. Comparison of a network in the asynchronous regime with a globally correlated external input (A) and a network in the synchronous regime with wave activity (B).** (**A**) The dynamics and activity statistics of a network with an added smoothly varying global signal, $\sigma_s s(t)$. Here, $s(t)$ is a shared source of smooth, unbiased Gaussian noise with auto-covariance function, $\text{cov}(s(t), s(t+\tau)) = \exp(-\tau^2/2\tau_s^2)$, where the correlation timescale is $\tau_s = 100$ms, and the magnitude of fluctuation is $\sigma_s = 0.5$ mV/ms. All the other parameters were the same as the default network. (**Aa**) The distribution of normalization indices in the network with added global signal (dashed line) and the default network (solid line). (**Ab**) Pairwise spike-count correlations are higher in the network with global noise than those in the default network. (**Ac**) Three consecutive spike raster snapshots, where a dot indicates that the excitatory neuron at the spatial position fired within 1 ms of the time stamp. (**Ad**) The magnitude of pairwise spike-count correlations in this network is similar to that in the data. Spike count correlations between neurons of similar normalization indices decrease with their average normalization indices, consistent with the data. (**Ae-g**) The normalization index of recurrent inhibitory current is strongly correlated with the firing rate normalization index, but that of recurrent excitatory current or feedforward excitatory current is not. Pearson correlation, **Ae**, $r = 0.27$, $p < 10^{-4}$; **Af**, $r = 0.22$, $p < 10^{-4}$; **Ag**, $r = -0.55$, $p < 10^{-4}$; $N = 14,364$. (**B**) Similar to (**A**), but for a network in the synchronous regime. The feedforward connection strength from V1 layer to inhibitory neurons in the V4/MT layer were $J_{\text{iF}} = 100$mV, compared to $J_{\text{iF}} = 140$mV in the default network model. All the other parameters were the same as the default network. (**Bc**) The network produces propagating wave dynamics. (**Bd**) Spike count correlations between neurons of similar normalization indices increase with their average normalization indices, inconsistent with the data. **Be-f**, Pearson correlation: **Be**, $r = 0.27$, $p < 10^{-4}$; **Bf**, $r = 0.24$, $p < 10^{-4}$; **Bg**, $r = -0.57$, $p < 10^{-4}$; $N = 16,333$.

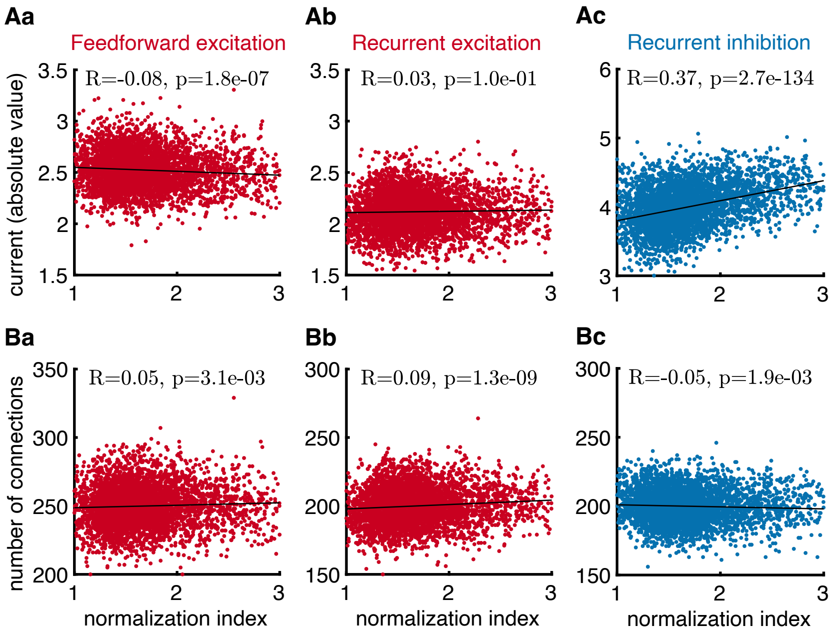

Figure S10: **Related to Fig. 3. The normalization index of neurons is strongly correlated with the magnitude of the mean recurrent inhibitory current.**

(**A**) There is a strong correlation between the firing rate normalization indexes and the average inhibitory current a neuron receives when two images are presented (**Ac**), and only weak correlations with the feedforward (**Aa**) and recurrent (**Ab**) excitatory currents. (**B**) The correlation between normalization and the number of excitatory or inhibitory input connections is weak.

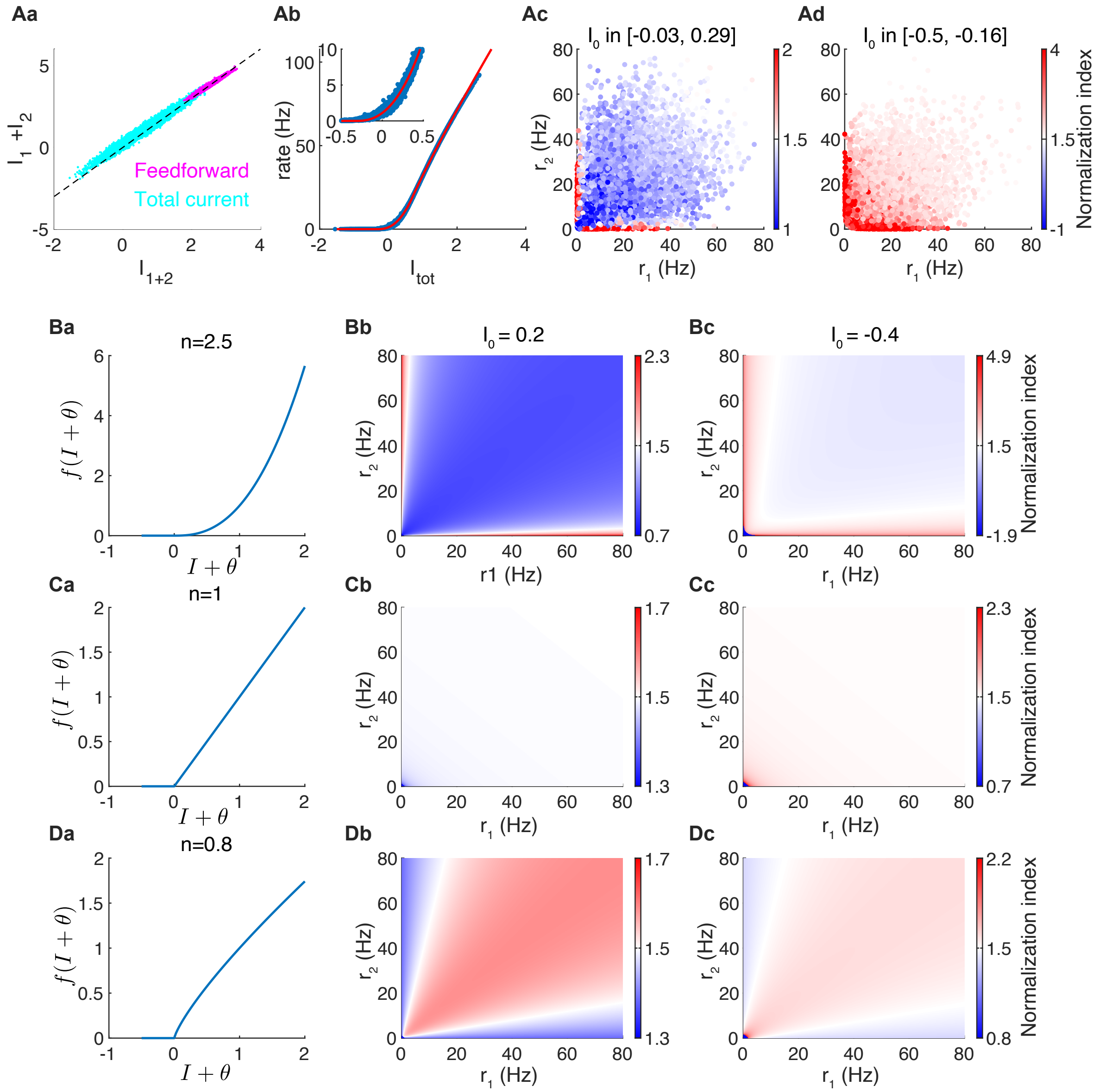

Figure S11: **Related to Fig. 3. The dependence of normalization index on neurons' firing rates when only stimulus 1 (r_1) or stimulus 2 (r_2) is presented in the default network model (A) and a feedforward model (B-D).** (**Aa**) The sum of feedforward current (magenta) or the total current (recurrent and feedforward, cyan) each neuron receives when only stimulus 1 (I_1) or stimulus 2 (I_2) is presented in our default network model compared to the current when both images are presented (I_{1+2}). Dashed line is where $I_{1+2} = (I_1 + I_2)/1.5$. (**Ab**) The transfer function of the excitatory neurons can be fitted with a rectified power-law function, $f(I) = \begin{cases} a \cdot [I-\theta]^n, I > \theta \\ 0, I \leq \theta \end{cases}$, with exponent $n \approx 2.45$ at low firing rates ($\leq$ 20 Hz) and $n = 0.87$ at high rates (> 20 Hz). Inset: zoom-in of the neurons around threshold. (**Ac**) Neurons with larger I_0 exhibit larger normalization indices if they selectively respond to one of the two stimuli. $I_0 = I_{1+2} - (I_1 + I_2)/1.5$. (**Ad**) Neurons with more negative I_0 generally exhibit larger normalization indices, especially when their responses to one of the stimuli are low. (**B-D**) The dependence of normalization index on r_1 and r_2 in a feedforward model, where $r_k = f(I_k)$, $k = \{1, 2, 1+2\}$ and $I_{1+2} = (I_1 + I_2)/1.5 + I_0$. The transfer function f is a rectified power law function with $n = 2.5$ (**Ba-Bc**), $n = 1$ (**Ca-Cc**), or $n = 0.8$ (**Da-Dc**). Color bars are centered at 1.5. The dependence of normalization index on r_1 and r_2 with a supralinear transfer function is consistent with those in the default network model. More details are in Supplementary Note 1.

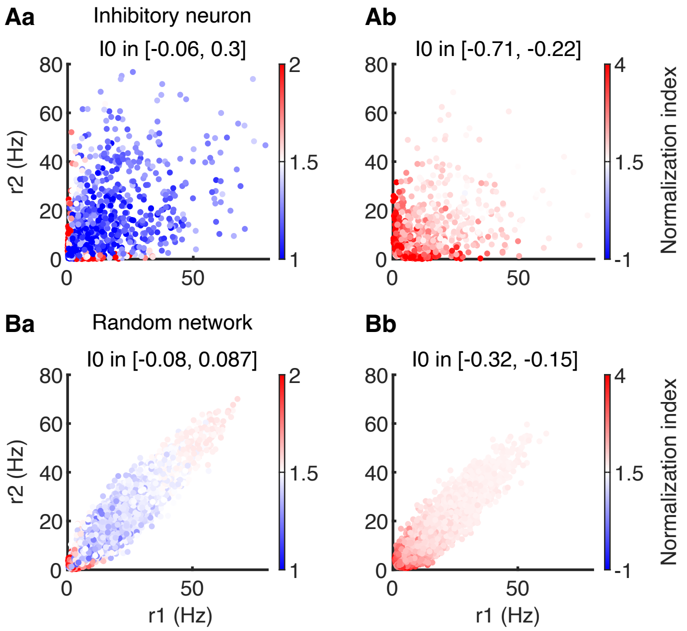

Figure S12: **Related to Fig. 3. The dependence of normalization index on neurons' firing rates when only stimulus 1 (r_1) or stimulus 2 (r_2) is presented for the inhibitory neurons in the default network model (A) or the excitatory neurons in a random network with disordered connections (B).** Same format as Figure S11Ac, Ad. The random network in **B** is the same as that in Figure S5 with overlap=1.

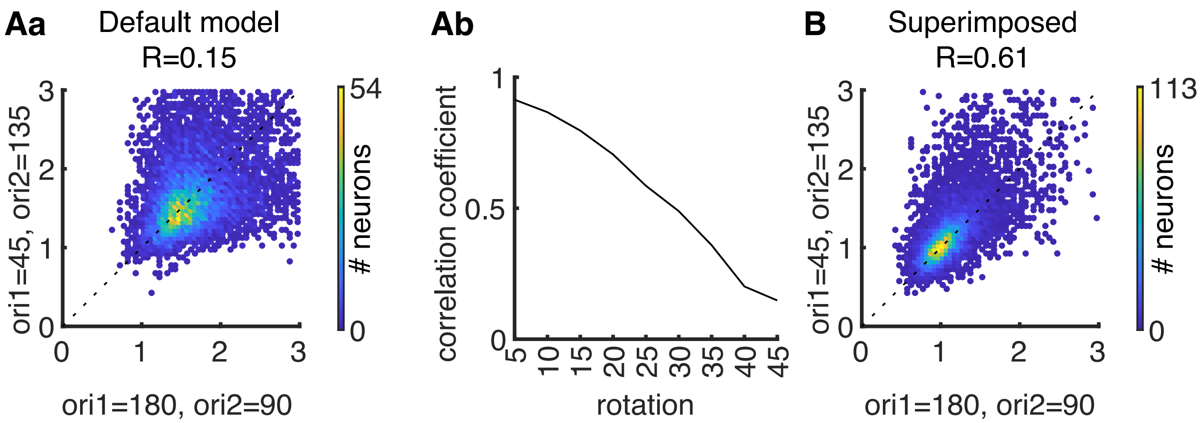

Figure S13: **Related to Fig. 3. The normalization strength varies with stimulus input.** (**Aa**) Normalization indices computed using the orientation pair (180°, 90°) show weak correlation with those computed using orientation pair (45°, 135°). Pearson correlation, $r = 0.15$, $p < 10^{-4}$, $N = 8,663$. (**Ab**) The orientations of the two images are rotated while keeping the two orientations orthogonal. The correlation between the normalization indices using the rotated orientations and those using the original pair (180°, 90°) decreases with the rotation degree. (**B**) Same as **Aa** with superimposed Gabor images. The normalization indices using orientation pair (180°, 90°) are highly correlated with those using orientation pair (45°, 135°), consistent with experimental findings from (*12*). Pearson correlation, $r = 0.61$, $p < 10^{-4}$, $N = 7,510$.

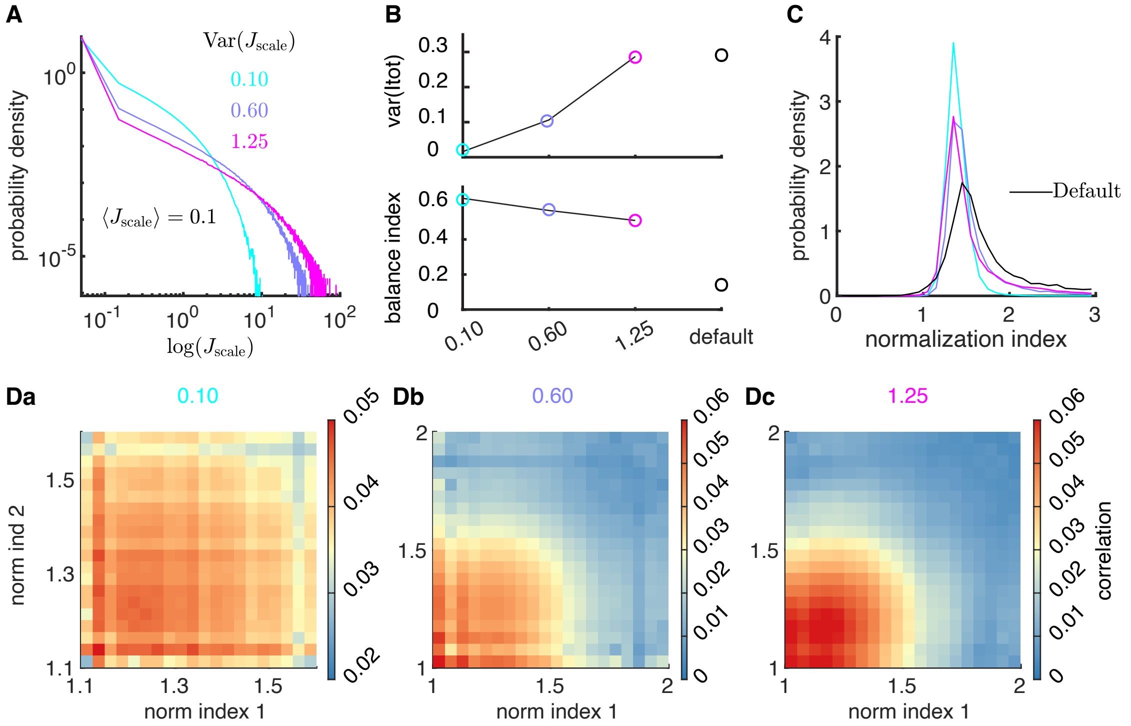

Figure S14: **Related to Fig. 2. Heterogeneous synaptic weights broaden the distribution of normalization indices.** The strength of each recurrent coupling is scaled by a factor J_{scale} that follows a Gamma distribution. (**A**) Distributions of J_{scale} with the same mean as 0.1 and different variances. (**B**) Top panel: as the variance of synaptic weights increases, the population variance of total input current increases and can be comparable to that from the default network (black circle). Bottom panel: the balance index decreases with Var(J_{scale}) and remains large. (**C**) The distribution of normalization indices broadens in networks with larger variance of weights. The distribution shifts toward lower normalization indices, which is different from that of the default network. (**Da**) The spike count correlations weakly depend on normalization indices when the variance of synaptic weights is low. (**Db-Dc**) The dependence of spike count correlations on normalization indices is consistent with data when the variance of synaptic weights is high. Other parameters are the same as those in the network with weak coupling (Figure S6, $J_{\text{scale}} = 0.1$ case).

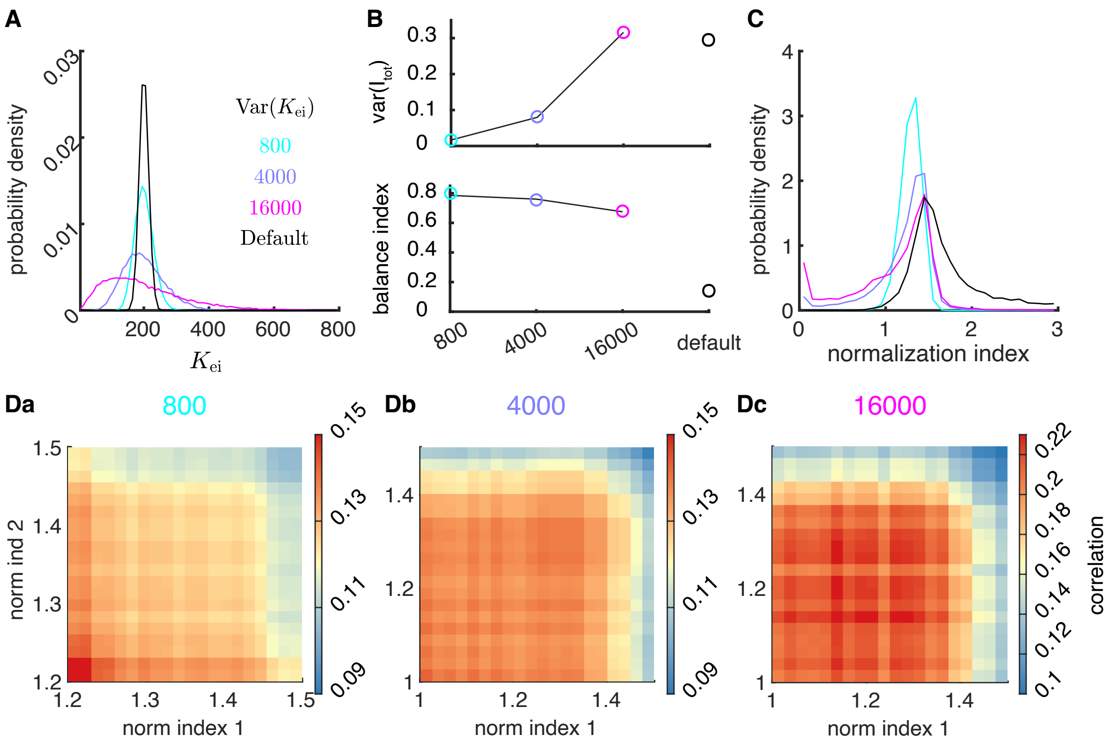

Figure S15: **Related to Fig. 2. Variability in synaptic in-degrees broadens the distribution of normalization indices, but does not reproduce the dependence of spike count correlation on normalization strength.** The recurrent synaptic weights are scaled by a factor of 0.1, meaning that the networks have weak coupling (same as in Figure S6, cyan). (**A**) The in-degrees of inhibitory-to-excitatory connections, K_{ei}, follows gamma distributions of different variances (800, 4000, 16000) and the same mean as in the default network. (**B**) Top panel: as $\mathrm{Var}(K_{ei})$ increases, the population variance of total input current increases and can be comparable to that from the default network (black circle). Bottom panel: the balance index decreases with $\mathrm{Var}(K_{ei})$ and remains large. (**C**) Larger variance of in-degrees broadens the distribution of normalization indices, which biases toward smaller index values. (**Da-Dc**) The spike count correlations weakly depend on normalization indices when the recurrent coupling is weak, despite large increases in in-degree variance. Other parameters are the same as those in the network with weak coupling (Figure S6, $J_{\mathrm{scale}} = 0.1$ case).

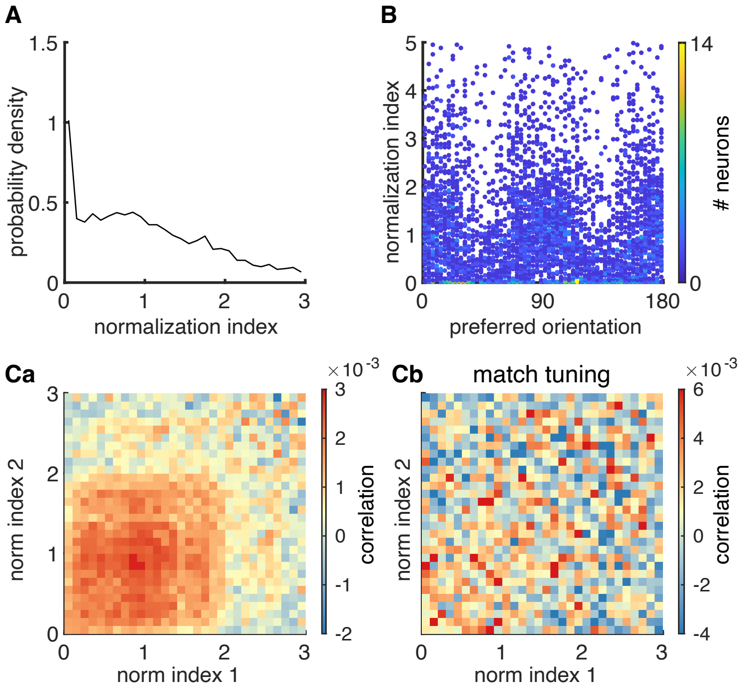

Figure S16: **The normalization index of a neuron strongly depends on the neuron's preferred orientation in the stabilized supralinear network (SSN) model.** The SSN model is a large-scale, probabilistically connected, 2D model of a visual area. E/I units are arranged on a grid of 75×75. Preferred orientations are assigned according to a superposed orientation map. Parameters of the model are the same as those used in Figure 6 of (*14*). Additionally, we apply globally correlated additive noise to each unit, ξ_i, where ξ_i satisfies $\tau_n d\xi_i = -\xi_i dt + \sigma_n(\sqrt{c}dW_0 + \sqrt{1-c}dW_i)$, with dW_0 and dW_i being Wiener process, $\tau_n = 40$ ms, $\sigma_n = 3.5$, and correlation $c = 0.2$. (**A**) The normalization index of model neurons is broadly distributed in the SSN model. (**B**) The normalization index and the tuning preference of model SSN neurons are statistically dependent (permutation test, $p = 0.001$). (**Ca**) Spike count correlations between a pair of neurons as a function of the normalization indexes of the pair. (**Cb**) The dependence of spike count correlation on normalization indexes is absent after matching for the distribution of tuning preferences of neurons across normalization indexes (**Cb**).

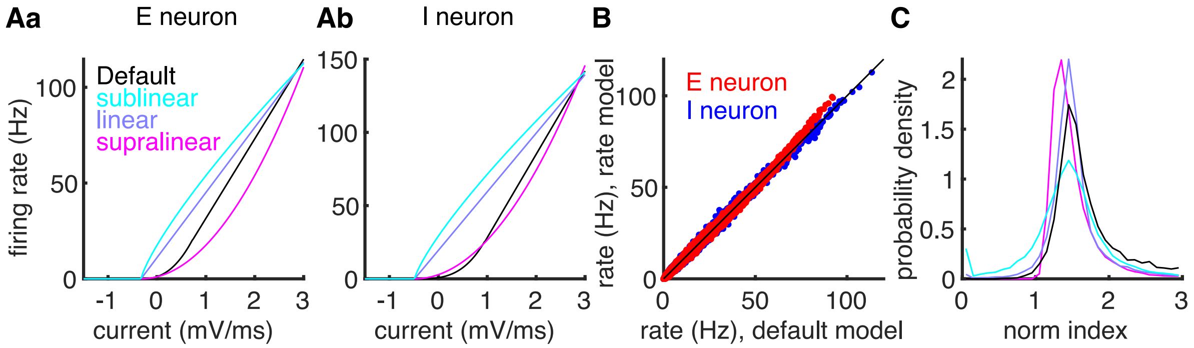

Figure S17: **Influence of the input-output transfer function on the distribution of normalization indices in recurrent networks.** (**Aa**) The fitted firing rate–current transfer function of the excitatory neurons from the default spiking network model (black, same as the red curve in Figure S11Ab), and three examples of rectified power-law transfer functions with different exponents: sublinear (n = 0.8), linear (n = 1), and supralinear (n = 2). All curves share the same threshold and dynamic range. (**Ab**) Same as **Aa** for the inhibitory neurons. (**B**) The rate model with fitted transfer functions (black curves in **Aa** and **Ab**) and the same connectivity matrix as in the spiking network model accurately reproduces the firing rates from the default spiking model for both excitatory (red) and inhibitory (blue) neurons. (**C**) The distributions of normalization indices in rate models with different transfer functions and the same connectivity matrix as that in the spiking network model. The model with a sublinear transfer function exhibits the largest heterogeneity in normalization indices. Equations for the rate model (Eq. S8) and the transfer function (Eq. S1) are in Supplementary Note 1.

REFERENCES

1. D. J. Heeger, Normalization of cell responses in cat striate cortex. *Vis. Neurosci.* **9**, 181–197 (1992).

2. T. Ohshiro, D. E. Angelaki, G. C. DeAngelis, A normalization model of multisensory integration. *Nat. Neurosci.* **14**, 775–782 (2011).

3. K. Louie, L. E. Grattan, P. W. Glimcher, Reward value-based gain control: Divisive normalization in parietal cortex. *J. Neurosci.* **31**, 10627–10639 (2011).

4. N. C. Rabinowitz, R. D. B. Willmore, J. W. Schnupp, A. J. King, Contrast gain control in auditory cortex. *Neuron* **70**, 1178–1191 (2011).

5. S. R. Olsen, V. Bhandawat, R. I. Wilson, Divisive normalization in olfactory population codes. *Neuron* **66**, 287–299 (2010).

6. D. Barbera, N. J. Priebe, L. L. Glickfeld, Feedforward mechanisms of cross-orientation interactions in mouse V1. *Neuron* **110**, 297–311.e4 (2022).

7. S. P. MacEvoy, T. R. Tucker, D. Fitzpatrick, A precise form of divisive suppression supports population coding in the primary visual cortex. *Nat. Neurosci.* **12**, 637–645 (2009).

8. A. M. Ni, S. Ray, J. H. R. Maunsell, Tuned normalization explains the size of attention modulations. *Neuron* **73**, 803–813 (2012).

9. M. Carandini, D. J. Heeger, Normalization as a canonical neural computation. *Nat. Rev. Neurosci.* **13**, 51–62 (2012).

10. J. H. Reynolds, D. J. Heeger, The normalization model of attention. *Neuron* **61**, 168–185 (2009).

11. J. Lee, J. H. Maunsell, A normalization model of attentional modulation of single unit responses. *PLOS ONE* **4**, e4651 (2009).

12. D. A. Ruff, J. J. Alberts, M. R. Cohen, Relating normalization to neuronal populations across cortical areas. *J. Neurophysiol.* **116**, 1375–1386 (2016).

13. B.-E. Verhoef, J. H. Maunsell, Attention-related changes in correlated neuronal activity arise from normalization mechanisms. *Nat. Neurosci.* **20**, 969–977 (2017).

14. D. B. Rubin, S. D. Van Hooser, K. D. Miller, The stabilized supralinear network: A unifying circuit motif underlying multi-input integration in sensory cortex. *Neuron* **85**, 402–417 (2015).

15. G. W. Lindsay, D. B. Rubin, K. D. Miller, A unified circuit model of attention: Neural and behavioral effects. bioRxiv 875534 [Preprint] (2019). https://doi.org/10.1101/2019.12.13.875534.

16. D. J. Heeger, W. E. Mackey, Oscillatory recurrent gated neural integrator circuits (organics), a unifying theoretical framework for neural dynamics. *Proc. Natl. Acad. Sci. U.S.A.* **116**, 22783–22794 (2019).

17. D. J. Heeger, K. O. Zemlianova, A recurrent circuit implements normalization, simulating the dynamics of V1 activity. *Proc. Natl. Acad. Sci. U.S.A.* **117**, 22494–22505 (2020).

18. O. Weiss, H. A. Bounds, H. Adesnik, R. Coen-Cagli, Modeling the diverse effects of divisive normalization on noise correlations. *PLoS Comput. Biol.* **19**, e1011667 (2023).

19. R. Coen-Cagli, S. S. Solomon, Relating divisive normalization to neuronal response variability. *J. Neurosci.* **39**, 7344–7356 (2019).

20. C. Huang, D. A. Ruff, R. Pyle, R. Rosenbaum, M. R. Cohen, B. Doiron, Circuit models of low-dimensional shared variability in cortical networks. *Neuron* **101**, 337–348.e4 (2019).

21. C. Van Vreeswijk, H. Sompolinsky, Chaos in neuronal networks with balanced excitatory and inhibitory activity. *Science* **274**, 1724–1726 (1996).

22. M. Kaschube, M. Schnabel, S. Löwel, D. M. Coppola, L. E. White, F. Wolf, Universality in the evolution of orientation columns in the visual cortex. *Science* **330**, 1113–1116 (2010).

23. J. Mariño, J. Schummers, D. C. Lyon, L. Schwabe, O. Beck, P. Wiesing, K. Obermayer, M. Sur, Invariant computations in local cortical networks with balanced excitation and inhibition. *Nat. Neurosci.* **8**, 194–201 (2005).

24. W. H. Bosking, Y. Zhang, B. Schofield, D. Fitzpatrick, Orientation selectivity and the arrangement of horizontal connections in tree shrew striate cortex. *J. Neurosci.* **17**, 2112–2127 (1997).

25. A. Angelucci, J. B. Levitt, E. J. S. Walton, J.-M. Hupe, J. Bullier, J. S. Lund, Circuits for local and global signal integration in primary visual cortex. *J. Neurosci.* **22**, 8633–8646 (2002).

26. R. Malach, Y. Amir, M. Harel, A. Grinvald, Relationship between intrinsic connections and functional architecture revealed by optical imaging and in vivo targeted biocytin injections in primate striate cortex. *Proc. Natl. Acad. Sci. U.S.A.* **90**, 10469–10473 (1993).

27. A. Renart, J. De La Rocha, P. Bartho, L. Hollender, N. Parga, A. Reyes, K. D. Harris, The asynchronous state in cortical circuits. *Science* **327**, 587–590 (2010).

28. A. Roxin, N. Brunel, D. Hansel, G. Mongillo, C. van Vreeswijk, On the distribution of firing rates in networks of cortical neurons. *J. Neurosci.* **31**, 16217–16226 (2011).

29. C. Huang, A. Pouget, B. Doiron, Internally generated population activity in cortical networks hinders information transmission. *Sci. Adv.* **8**, eabg5244 (2022).

30. D. L. Ringach, R. M. Shapley, M. J. Hawken, Orientation selectivity in macaque V1: Diversity and laminar dependence. *J. Neurosci.* **22**, 5639–5651 (2002).

31. M. R. Cohen, J. H. R. Maunsell, Attention improves performance primarily by reducing interneuronal correlations. *Nat. Neurosci.* **12**, 1594–1600 (2009).

32. Y. Gu, S. Liu, C. R. Fetsch, Y. Yang, S. Fok, A. Sunkara, G. C. DeAngelis, D. E. Angelaki, Perceptual learning reduces interneuronal correlations in macaque visual cortex. *Neuron* **71**, 750–761 (2011).

33. A. Ponce-Alvarez, A. Thiele, T. D. Albright, G. R. Stoner, G. Deco, Stimulus-dependent variability and noise correlations in cortical MT neurons. *Proc. Natl. Acad. Sci. U.S.A.* **110**, 13162–13167 (2013).

34. I.-C. Lin, M. Okun, M. Carandini, K. D. Harris, The nature of shared cortical variability. *Neuron* **87**, 644–656 (2015).

35. G. Hennequin, Y. Ahmadian, D. B. Rubin, M. Lengyel, K. D. Miller, The dynamical regime of sensory cortex: Stable dynamics around a single stimulus-tuned attractor account for patterns of noise variability. *Neuron* **98**, 846–860.e5 (2018).

36. L. Busse, A. R. Wade, M. Carandini, Representation of concurrent stimuli by population activity in visual cortex. *Neuron* **64**, 931–942 (2009).

37. G. K. Ocker, Y. Hu, M. A. Buice, B. Doiron, K. Josić, R. Rosenbaum, E. Shea-Brown, From the statistics of connectivity to the statistics of spike times in neuronal networks. *Curr. Opin. Neurobiol.* **46**, 109–119 (2017).

38. M. R. Cohen, A. Kohn, Measuring and interpreting neuronal correlations. *Nat. Neurosci.* **14**, 811–819 (2011).

39. N. Brunel, Dynamics of sparsely connected networks of excitatory and inhibitory spiking neurons. *J. Comput. Neurosci.* **8**, 183–208 (2000).

40. N. Mosheiff, B. Ermentrout, C. Huang, Chaotic dynamics in spatially distributed neuronal networks generate population-wide shared variability. *PLoS Comput. Biol.* **19**, e1010843 (2023).

41. P. Seriès, P. E. Latham, A. Pouget, Tuning curve sharpening for orientation selectivity: Coding efficiency and the impact of correlations. *Nat. Neurosci.* **7**, 1129–1135 (2004).

42. A. Kohn, R. Coen-Cagli, I. Kanitscheider, A. Pouget, Correlations and neuronal population information. *Annu. Rev. Neurosci.* **39**, 237–256 (2016).

43. J. Beck, V. R. Bejjanki, A. Pouget, Insights from a simple expression for linear fisher information in a recurrently connected population of spiking neurons. *Neural Comput.* **23**, 1484–1502 (2011).

44. I. Kanitscheider, R. Coen-Cagli, A. Kohn, A. Pouget, Measuring fisher information accurately in correlated neural populations. *PLoS Comput. Biol.* **11**, e1004218 (2015).

45. S. Chung, D. D. Lee, H. Sompolinsky, Classification and geometry of general perceptual manifolds. *Phys. Rev. X* **8**, 031003 (2018).

46. C. Stephenson, J. Feather, S. Padhy, O. Elibol, H. Tang, J. McDermott, S. Chung, Untangling in invariant speech recognition. *Adv. Neural. Inf. Process Syst.* **32**, 14391–14401 (2019).

47. K. H. Britten, H. W. Heuer, Spatial summation in the receptive fields of MT neurons. *J. Neurosci.* **19**, 5074–5084 (1999).

48. N. C. Rust, V. Mante, E. P. Simoncelli, J. A. Movshon, How MT cells analyze the motion of visual patterns. *Nat. Neurosci.* **9**, 1421–1431 (2006).

49. S.-C. Guan, S.-H. Zhang, Y.-C. Zhang, S.-M. Tang, C. Yu, Plaid detectors in macaque V1 revealed by two-photon calcium imaging. *Curr. Biol.* **30**, 934–940.e3 (2020).

50. A. Sanzeni, A. Palmigiano, T. H. Nguyen, J. Luo, J. J. Nassi, J. H. Reynolds, M. H. Histed, K. D. Miller, N. Brunel, Mechanisms underlying reshuffling of visual responses by optogenetic stimulation in mice and monkeys. *Neuron* **111**, 4102–4115.e9 (2023).

51. M. Okun, I. Lampl, Instantaneous correlation of excitation and inhibition during ongoing and sensory-evoked activities. *Nat. Neurosci.* **11**, 535–537 (2008).

52. B. Haider, A. Duque, A. R. Hasenstaub, D. A. McCormick, Neocortical network activity in vivo is generated through a dynamic balance of excitation and inhibition. *J. Neurosci.* **26**, 4535–4545 (2006).

53. M. Xue, B. V. Atallah, M. Scanziani, Equalizing excitation–inhibition ratios across visual cortical neurons. *Nature* **511**, 596–600 (2014).

54. C. van Vreeswijk, H. Sompolinsky, Chaotic balanced state in a model of cortical circuits. *Neural Comput.* **10**, 1321–1371 (1998).

55. C. Baker, V. Zhu, R. Rosenbaum, Nonlinear stimulus representations in neural circuits with approximate excitatory-inhibitory balance. *PLoS Comput. Biol.* **16**, e1008192 (2020).

56. M. Carandini, D. J. Heeger, J. A. Movshon, Linearity and normalization in simple cells of the macaque primary visual cortex. *J. Neurosci.* **17**, 8621–8644 (1997).

57. F. S. Chance, L. F. Abbott, A. D. Reyes, Gain modulation from background synaptic input. *Neuron* **35**, 773–782 (2002).

58. T. K. Sato, B. Haider, M. Häusser, M. Carandini, An excitatory basis for divisive normalization in visual cortex. *Nat. Neurosci.* **19**, 568–570 (2016).

59. N. J. Priebe, D. Ferster, Mechanisms underlying cross-orientation suppression in cat visual cortex. *Nat. Neurosci.* **9**, 552–561 (2006).

60. G. Mongillo, S. Rumpel, Y. Loewenstein, Inhibitory connectivity defines the realm of excitatory plasticity. *Nat. Neurosci.* **21**, 1463–1470 (2018).

61. J. H. Reynolds, L. Chelazzi, R. Desimone, Competitive mechanisms subserve attention in macaque areas V2 and V4. *J. Neurosci.* **19**, 1736–1753 (1999).

62. S. Treue, J. H. Maunsell, Attentional modulation of visual motion processing in cortical areas MT and MST. *Nature* **382**, 539–541 (1996).

63. A. Thiele, C. Brandt, M. Dasilva, S. Gotthardt, D. Chicharro, S. Panzeri, C. Distler, Attention induced gain stabilization in broad and narrow-spiking cells in the frontal eye-field of macaque monkeys. *J. Neurosci.* **36**, 7601–7612 (2016).

64. J. F. Mitchell, K. A. Sundberg, J. H. Reynolds, Differential attention-dependent response modulation across cell classes in macaque visual area V4. *Neuron* **55**, 131–141 (2007).

65. T. Kanashiro, G. K. Ocker, M. R. Cohen, B. Doiron, Attentional modulation of neuronal variability in circuit models of cortex. *eLife* **6**, e23978 (2017).

66. D. C. Somers, E. V. Todorov, A. G. Siapas, L. J. Toth, D.-S. Kim, M. Sur, A local circuit approach to understanding integration of long-range inputs in primary visual cortex. *Cereb. Cortex* **8**, 204–217 (1998).

67. A. Angelucci, M. Bijanzadeh, L. Nurminen, F. Federer, S. Merlin, P. C. Bressloff, Circuits and mechanisms for surround modulation in visual cortex. *Annu. Rev. Neurosci.* **40**, 425–451 (2017).

68. A. J. Keller, M. Dipoppa, M. M. Roth, M. S. Caudill, A. Ingrosso, K. D. Miller, M. Scanziani, A disinhibitory circuit for contextual modulation in primary visual cortex. *Neuron* **108**, 1181–1193.e8 (2020).

69. A. Tran-Van-Minh, R. D. Cazé, T. Abrahamsson, L. Cathala, B. S. Gutkin, D. A. DiGregorio, Contribution of sublinear and supralinear dendritic integration to neuronal computations. *Front. Cell. Neurosci.* **9**, 67 (2015).

70. G. Turrigiano, Too many cooks? Intrinsic and synaptic homeostatic mechanisms in cortical circuit refinement. *Annu. Rev. Neurosci.* **34**, 89–103 (2011).

71. D. A. Kaliukhovich, R. Vogels, Divisive normalization predicts adaptation-induced response changes in macaque inferior temporal cortex. *J. Neurosci.* **36**, 6116–6128 (2016).

72. F. Höfflin, A. Jack, C. Riedel, J. Mack-Bucher, J. Roos, C. Corcelli, C. Schultz, P. Wahle, M. Engelhardt, Heterogeneity of the axon initial segment in interneurons and pyramidal cells of rodent visual cortex. *Front. Cell. Neurosci.* **11**, 332 (2017).

73. F. Rieke, M. E. Rudd, The challenges natural images pose for visual adaptation. *Neuron* **64**, 605–616 (2009).

74. O. Schwartz, E. P. Simoncelli, Natural signal statistics and sensory gain control. *Nat. Neurosci.* **4**, 819–825 (2001).

75. M. Di Volo, A. Destexhe, Optimal responsiveness and information flow in networks of heterogeneous neurons. *Sci. Rep.* **11**, 17611 (2021).

76. A. Hutt, S. Rich, T. A. Valiante, J. Lefebvre, Intrinsic neural diversity quenches the dynamic volatility of neural networks. *Proc. Natl. Acad. Sci. U.S.A.* **120**, e2218841120 (2023).

77. T. O. Sharpee, Optimizing neural information capacity through discretization. *Neuron* **94**, 954–960 (2017).

78. D. B. Kastner, S. A. Baccus, T. O. Sharpee, Critical and maximally informative encoding between neural populations in the retina. *Proc. Natl. Acad. Sci.* **112**, 2533–2538 (2015).

79. N. Perez-Nieves, V. C. Leung, P. L. Dragotti, D. F. Goodman, Neural heterogeneity promotes robust learning. *Nat. Commun.* **12**, 5791 (2021).

80. I. Kanitscheider, R. Coen-Cagli, A. Pouget, Origin of information-limiting noise correlations. *Proc. Natl. Acad. Sci. U.S.A.* **112**, E6973–E6982 (2015).

81. A. Kohn, M. A. Smith, Stimulus dependence of neuronal correlation in primary visual cortex of the macaque. *J. Neurosci.* **25**, 3661–3673 (2005).

82. M. Kafashan, A. W. Jaffe, S. N. Chettih, R. Nogueira, I. Arandia-Romero, C. D. Harvey, R. Moreno-Bote, J. Drugowitsch, Scaling of sensory information in large neural populations shows signatures of information-limiting correlations. *Nat. Commun.* **12**, 473 (2021).

83. R. Moreno-Bote, J. Beck, I. Kanitscheider, X. Pitkow, P. Latham, A. Pouget, Information-limiting correlations. *Nat. Neurosci.* **17**, 1410–1417 (2014).

84. Y. Ahmadian, K. D. Miller, What is the dynamical regime of cerebral cortex? *Neuron* **109**, 3373–3391 (2021).
